# NFAT5 Controls the Integrity of Epidermis 

**DOI:** 10.3389/fimmu.2021.780727

**Published:** 2021-12-09

**Authors:** Khalid Muhammad, Delicia Xavier, Stefan Klein-Hessling, Muhammad Azeem, Tabea Rauschenberger, Krisna Murti, Andris Avots, Matthias Goebeler, Matthias Klein, Tobias Bopp, Malte Sielaff, Stefan Tenzer, Sigrid Möckel, José Aramburu, Cristina López-Rodríguez, Andreas Kerstan, Edgar Serfling

**Affiliations:** ^1^ Department of Molecular Pathology, Institute of Pathology, University of Wuerzburg, Wuerzburg, Germany; ^2^ Comprehensive Cancer Centre Mainfranken, Wuerzburg, Germany; ^3^ Department of Dermatology, Venereology and Allergology, University Hospital Wuerzburg, Wuerzburg, Germany; ^4^ Institute for Immunology, University Medical Center, University of Mainz, Mainz, Germany; ^5^ Research Center for Immunotherapy, University Medical Center, University of Mainz, Mainz, Germany; ^6^ University Cancer Center Mainz, University Medical Center, University of Mainz, Mainz, Germany; ^7^ Institute of Pathology, University of Wuerzburg, Wuerzburg, Germany; ^8^ Immunology Unit, Department of Experimental and Health Sciences, Universitat Pompeu Fabra, Barcelona, Spain

**Keywords:** epidermis, keratinocytes, kallikrein 7, matrix proteases, Mmp3, NFAT5, skin

## Abstract

The skin protects the human body against dehydration and harmful challenges. Keratinocytes (KCs) are the most abundant epidermal cells, and it is anticipated that KC-mediated transport of Na^+^ ions creates a physiological barrier of high osmolality against the external environment. Here, we studied the role of NFAT5, a transcription factor whose activity is controlled by osmotic stress in KCs. Cultured KCs from adult mice were found to secrete more than 300 proteins, and upon NFAT5 ablation, the secretion of several matrix proteinases, including metalloproteinase-3 (Mmp3) and kallikrein-related peptidase 7 (Klk7), was markedly enhanced. An increase in Mmp3 and Klk7 RNA levels was also detected in transcriptomes of *Nfat5^-/-^* KCs, along with increases of numerous members of the ‘Epidermal Differentiation Complex’ (EDC), such as small proline-rich (Sprr) and S100 proteins. NFAT5 and Mmp3 as well as NFAT5 and Klk7 are co-expressed in the basal KCs of fetal and adult epidermis but not in basal KCs of newborn (NB) mice. The poor NFAT5 expression in NB KCs is correlated with a strong increase in Mmp3 and Klk7 expression in KCs of NB mice. These data suggests that, along with the fragile epidermis of adult *Nfat5^-/-^* mice, NFAT5 keeps in check the expression of matrix proteases in epidermis. The NFAT5-mediated control of matrix proteases in epidermis contributes to the manifold changes in skin development in embryos before and during birth, and to the integrity of epidermis in adults.

## Introduction

The skin represents a physical barrier that shields the body against harmful environmental challenges and protects against dehydration (1). Large amounts of Na^+^ ions are stored in the skin, and it has been anticipated that an active transport of Na^+^ by KCs creates a physiological barrier within or below the epidermis with high osmolality (2–4). One factor that could orchestrate the storage of salt ions in the skin is the osmo-sensitive transcription factor NFAT5. However, so far there are no detailed studies on the function of NFAT5 in the skin.

To a large part, the skin barrier formation and function are conferred by the corneocytes in the *stratum corneum*, the outermost epidermal layer. In healthy adult epidermis, corneocytes are terminally differentiated KCs that assemble an insoluble complex of lipids and numerous adhesion proteins which are organized in corneodesmosomes (5, 6). Extracellular matrix proteases, such as metalloproteases and kallikreins, are secreted by KCs, cleave corneodesmosomes and, thereby, release corneocytes as skin scales (7, 8).

The differentiation of basal epidermal KCs to corneocytes is a fine-tuned process that lasts approximately 3-4 weeks in human and one week in murine skin. Only KCs of the *stratum basale* (the basal KCs) proliferate and, when detached, differentiate into cells of the *stratum spinosum*, followed by development to the *stratum granulosum*, and finally to the corneocytes of the *stratum corneum*. This well-coordinated Ca^++^-dependent differentiation process is controlled by the activity of numerous genes that are specifically expressed in various epidermal layers.

Genes that are expressed predominantly or exclusively in the basal layer are several keratin, laminin and integrin genes. The K5/14 keratin heterodimers encoded by the keratin *Krt5* and *Krt14* genes constitute almost 50% of the protein content of basal KCs. Rising Ca^++^ levels in the upper epidermal layers promote differentiation. The synthesis of K5/14 keratins ceases and shifts to the expression of K1/10 keratin heterodimers (9–11). The laminin subunits gamma-2 (LAMC2) and beta-3 (LAMB3) as well as the α3β1 and α6β4 integrin heterodimers support basal KCs to assemble and to adhere to the basement membrane. Their synthesis is inhibited with the onset of differentiation, and in case of integrins, is replaced by other members of the integrin family (12).

We show here that the osmo-sensitive transcription factor NFAT5 (13) controls the transition of basal to suprabasal KCs, and finally to corneocytes in adult skin. While we did not detect an obvious role for NFAT5 in the intracellular transport of Na^+^ ions in or through the epidermis, NFAT5 controls the expression of numerous genes that orchestrate the differentiation of KCs to corneocytes. In basal KCs, NFAT5 suppresses differentiation by inhibiting the formation of ‘cornification proteins’, such as Sprr and S100 proteins and the transglutaminases Tgm1 and Tgm2. The suppression of matrix proteases kallikrein 7 (Klk7) and matrix metallopeptidase 3 (Mmp3) in basal KCs of adult mice contributes to the integrity of skin that, upon NFAT5 ablation, shows a pre-mature shedding of skin scales. By contrast, both matrix proteases are 10-fold stronger generated in KCs from NB mice in which NFAT5 is poorly expressed. Similar to their coordinated expression in adult KCs, NFAT5 and Mmp3 are simultaneously expressed in KCs of murine and human embryos suggesting a role for NFAT5 in early skin formation and later on in the integrity of adult skin.

## Materials and Methods

### Mice, Preparation and Short-Term Cultures of Primary Murine KCs and T Cells

If not stated otherwise, 8- to 12-week-old C57BL/6J mice were used in the experiments. All *Nfat5^-/-^* mice were on the 129/sv background, and in all assays in which they were used littermate WT mice were analysed. 129sv mice are smaller in size, show a kidney atrophy and are under permanent hypernatremia (14, 15). For the preparation of NFAT5-deficient KCs, the tails from *Nfat5^-/-^* and their wild type (WT) siblings were sent from Barcelona to Würzburg overnight in wet chambers on ice and used for the preparation of KCs. Primary KCs from tails of adult mice or from NB mice were prepared as described (16). However, instead of 0.25% porcine trypsin that in our hands killed primary murine KCs, TrypLE (ThermoFisher) was used for the preparation and splitting of cells. The skin of mice was incubated overnight in dispase solution (4 mg/ml, in KC medium) at 4°C. The epidermis was prepared, divided into small pieces and incubated in TrypLE for 15 min at 37°C. After suspension in S-MEM medium (Sigma-Aldrich), strong shaking and passage through a 70 μM cell strainer, the cells were plated on pre-coated plates for 5 h, followed by a PBS wash step and incubation in serum-free SFM KC medium (Gibco) containing 0.06 mM Ca^++^.

Splenic murine CD4^+^T cells were prepared as described previously, and cultivated in RPMI medium. They were activated either by antiCD3/CD28 (3/1 μg/ml), coated on plastic, or TPA (10 ng/ml) plus ionomycin (0.5 μM) for 24 h.

### Transduction of KCs With NFAT5-Bio Retroviruses

Retroviruses expressing tagged NFAT5-bio and GFP were generated by transfections of plat E packaging cells (17) with viral NFAT5-bio/GFP or GFP constructs using GenJet Reagent (SigmaGen Laboratories). The next day, the medium was changed, and the following day the supernatant of plat E cells was passed through a 0.45 μm filter and, upon incubation with polybrene (4 μl per ml of a 1 mg/ml stock solution) for 20 min at room temperature, used in spin transfections. KCs grown in 2 ml SFM KC medium on pre-coated 6-well plates were transduced by adding 2 ml retroviral supernatant to 1 ml of culture medium, followed by centrifugation of parafilm-sealed culture plates at 32°C and 2200 rpm for 2.5 h. After centrifugation, the plates were maintained for 2 h at 37°C in a CO_2_ incubator, followed by washing of cells with PBS and culture in KC medium. The efficiency of transduction was monitored by fluorescence microscopy and by transducing NIH 3T3 control cells in parallel, which revealed GFP expression in more than 60% of the cells by flow cytometry (see **Figure 5E**).

For construction of the NFAT5-bio vector, full-length human NFAT5 (18) was amplified by PCR using the primers 5`Cla_hNFAT5: 5`ttatcgatggcggtgcttgcagctcc3` and 3`hNFAT5_Bio: 5`cagacctccaccgcccaattgaaaggagccagtcaagttg3`. The bio/avidin-tag (19) was generated by PCR using the primers 5`hNFAT5C_Bio: 5`caacttgactggctcctttcaattgggcggtggaggtctg3` and 3`BamHI_Bio: 5`aaggatcccacgagcctccggcgtttgag3`. The overlapping products were amplified by a second PCR using the primers 5`Cla-hNFAT5 and 3`BamHI_Bio and cloned as a ClaI/BamHI fragment into the retroviral expression vector pEGZ (20). The retroviral pMSCV-F-BirA vector was purchased from BCCM/LMBP (Gent-Zwijnaarde, Belgium).

### Secretion Assays

To determine the secretome of WT and *Nfat5^-/-^* KCs, KC proteins were labelled with azidohomoalanine (AHA) for 20 h, followed by click chemistry-based enrichment of secreted proteins, on-resin trypsinisation and liquid chromatography-mass spectrometry (LC-MS) analysis (21). To this end, 2-3 x10^6^ KCs maintained in a 10-cm culture dish for one week *in vitro* were incubated with 7 ml RPMI-depletion medium (lacking methionine, arginine and lysine) for 30 min, followed by incubation for 20 h in methionine-free RPMI supplemented with lysine, arginine and 0.1 mM AHA, an azide-bearing analog of methionine. The cells were harvested, washed three times with PBS and quickly frozen in liquid nitrogen upon adding protease inhibitor.

### Enrichment of Secreted Proteins and Quantitative Proteomic Analysis

Azide-tagged proteins were enriched from cell culture supernatants of primary KCs isolated from wild type and *Nfat5^-/-^* mice using a click chemistry approach according to manufacturer’s instructions (Click Chemistry Capture Kit, Jena Bioscience, Jena, Germany) with minor modifications. Briefly, 7 ml of media were concentrated on a centrifugal filter unit (Amicon Ultra-15, 3 kDa MWCO, Merck, Darmstadt, Germany) at 4,000*g* and 4°C to a final volume of 250 µl. The click reaction was assembled in a 2 ml tube (Protein LoBind tubes, Eppendorf, Hamburg, Germany) by combining the concentrated sample, 250 µl of lysis buffer (200 mM Tris, 4% CHAPS, 1 M NaCl, 8 M urea, pH 8.0), 500 µl of 2× copper catalyst solution and 100 µl of washed alkyne agarose resin (Jena Bioscience). The reaction was incubated on a rotator at room temperature for 20 h. After reduction and alkylation of resin-bound proteins using dithiothreitol (DTT) and iodoacetamide (IAA), the resin was transferred to a spin column. The column was washed five times using 2 ml of agarose wash buffer (100 mM Tris, 1% SDS, 250 mM NaCl, 5 mM EDTA, pH 8.0), ten times 2 ml of urea buffer (100 mM Tris, 8 M urea, pH 8.0) and 10 times 2 ml of 20% (v/v) LC-MS grade acetonitrile/water. The resin was recovered in digestion buffer (50 mM ammonium bicarbonate and LC-MS grade water), transferred to a new tube and centrifuged at 1,000g for 5 min. The supernatant was discarded, 0.5 µg of trypsin (Trypsin Gold, Promega, Madison, WI, USA) was added to the remaining 200 µl of resin slurry and the samples were incubated overnight at 37°C. Afterwards, the resin was pelleted and the supernatant containing tryptic peptides was transferred to a clean tube. To improve peptide recovery, the resin was resuspended in 500 µl of LC-MS grade water. After centrifugation, the supernatant was transferred to the digest supernatant. Peptides were acidified with trifluoroacetic acid (TFA) to achieve a final concentration of 0.5% (v/v) and desalted on Sep-Pak tC18 96-well cartridges (Waters Corporation, Milford, MA, USA) using 0.1% (v/v) TFA in LC-MS grade water as wash solvent and 0.1% (v/v) TFA in 50% (v/v) acetonitrile/water as elution solvent. Purified peptides were lyophilized and reconstituted in 20 µl of 0.1% (v/v) formic acid in LC-MS grade water prior LC-MS analysis. To control for non-specifically enriched proteins, one-tenth of the cell culture supernatant was separated and processed in parallel in a similar manner, but without adding copper sulfate.

Peptide samples were analysed by consecutive LC-MS runs in triplicates using a nanoACQUITY UPLC system (Waters Corporation, Milford, MA, USA) coupled to a SYNAPT G2-S mass spectrometer (Waters Corporation) *via* a NanoLockSpray dual electrospray ionization source (Waters Corporation). Equal sample volumes of 0.3 µl were injected onto a HSS-T3 C18 250 mm × 750 µm reversed phase column (Waters Corporation) for each measurement and peptides separated by gradient elution over 90 min resulting in total analysis times of 110 min. Mass spectra were acquired in ion mobility-enhanced data-independent mode (UDMSE) (PMID: 24336358). [For details see (22)].

Raw data were processed by ProteinLynx Global Server (v3.0.2, Waters Corporation) and searched against the mouse Swiss-Prot protein sequence database (UniProtKB release 2018_07, 16,669 entries) and 171 common MS contaminants using following parameters: Trypsin was specified as digestion enzyme, two missed cleavages per peptide were allowed, carbamidomethylation of cysteines was set as fixed, and methionine oxidation as variable modification. The false discovery rate (FDR) was calculated in PLGS by searching a database of reversed protein sequences and a cutoff of 0.01 was applied. Label-free quantification (LFQ) including retention time alignment, feature clustering, cross-run normalization and protein inference was performed in ISOQuant v1.8 (22). At this time, control samples were not included in the analysis to avoid normalization artefacts. Only peptides without missed cleavages, a minimum sequence length of six amino acids, a minimum PLGS score of 6.0 and no variable modification were considered for quantification. Proteins identified by at least two different peptides were quantified by averaging the intensities of the three peptides with the highest intensities belonging to the respective protein (Top3 method, https://doi.org/10.1074/mcp.M500230-MCP200). An FDR cutoff of 0.01 was applied at the peptide and protein level in ISOQuant. The ISOQuant analysis was repeated without normalization including control samples in order to identify non-specifically enriched proteins. Proteins found in more than one biological replicate with at least one-tenth of the Top3 intensity in the negative control compared with the sample were not considered potential candidates.

Differential protein abundance testing between wild type and *Nfat5^-/-^* animals was performed using Perseus v1.6.2.1 (PMID: 27348712) following log_2_-transformation of Top3 intensities and left-censored missing value imputation (“replace missing values from normal distribution” with default settings). *P*-values derived from two-sided Student’s *t*-tests were adjusted for multiple hypothesis testing using the Benjamini-Hochberg method applying an FDR cutoff of 0.05.

The MS proteomics data have been deposited to the ProteomeXchange Consortium *via* the PRIDE partner repository with the dataset identifier PXD028675 (Project name: Murine keratinocytes NFAT5 knockout secretome LC-MS/MS).

### Western Blot and RT-PCR Assays

Western blots were performed either with whole protein extracts or with extracts of cytosolic and nuclear proteins on PAGE-SDS gels. For detecting NFAT5, the following Abs were used: Ab3446 (directed against the C-terminal aa1439-1455 of human NFAT5), Ab110995 (directed against an internal region of human NFAT5; both Abcam), sc-398171 (directed against aa67-300 of human NFAT5; Santa Cruz, Biotech) and PAI-023 (directed against a C-terminal peptide of human NFAT5; Affinity BioReagents). NFATc proteins were detected using the 7A6 mAb #556602 (for NFATc1) and the mAb #5062574 (for NFATc2; both BD Pharmingen). As loading control, filters were stained by Ponceau Red for 5 min and/or re-probed with the mAb #ab8227 specific for β-actin. Signals were developed using a chemiluminescence detection system (ThermoFisher Scientific).

For qRT-PCR assays, RNA was isolated from freshly harvested and PBS-washed or from deep-frozen cells using a standard TRIzol/isopropanol protocol. cDNAs were synthesized using the iScript cDNA synthesis kit according to the manufacturer’s instructions (Bio-Rad). Real-time PCR assays were performed using the SYBR green master mix (Applied Biosystems) with the primers presented in **Supplementary Table 1**.

### Transcriptome Assays by Next Generation Sequencing (NGS)

Total RNA was purified with RNeasy Plus Micro Kit according to the manufacturer’s protocol (Qiagen) and quantified using a Qubit 2.0 fluorometer (Invitrogen). Quality was assessed on a Bioanalyzer 2100 (Agilent) using a RNA 6000 Nano chip (Agilent). Samples with an RNA integrity number (RIN) of > 8 were used for library preparation. Barcoded mRNA-seq cDNA libraries were prepared from 600 ng (WT vs Nfat5-KO experiment) or 140 ng (1 week vs 3 weeks experiment) of total RNA using the NEBNext^®^ Poly(A) mRNA Magnetic Isolation Module and NEBNext^®^ Ultra™ II RNA Library Prep Kit for Illumina^®^ according to the manual with a final amplification of 11 (WT vs Nfat5) or 12 PCR cycles (1 week vs. 3 weeks). The quantity was assessed using Invitrogen’s Qubit HS DNA assay kit. The library size was determined using Agilent’s 2100 Bioanalyzer HS DNA assay. Barcoded RNA-Seq libraries were onboard clustered using HiSeq^®^ Rapid SR Cluster Kit v2 using 8pM and 59bps were sequenced on the Illumina HiSeq2500 using HiSeq^®^ Rapid SBS Kit v2 (59 cycles). The raw output data of the HiSeq was pre-processed according to the Illumina standard protocol. Sequence reads were trimmed for adapter sequences and further processed using Qiagen’s software CLC Genomics Workbench (v12.0 with CLC’s default settings for RNA-Seq analysis). Reads were aligned to the GRCm38 genome and expression values are RPKM. - All sequencing data were submitted to the GEO repository and are publicly available (https://www.ncbi.nlm.nih.gov/geo/query/acc.cgi?acc=GSE184180).

### Immunohistochemistry and Tape Stripping Assays

Primary KCs were fixed with 4% formaldehyde (in PBS) on culture dishes followed by incubation with 0.2% Triton X-100. After PBS washing, the cells were blocked with DAKO diluent solution followed by incubation for 90 min with primary Abs (diluted 1:100-1:400) in DAKO blocking solution. The primary Abs used are indicated in the figure legends.

Anonymized tissue specimens of human fetal skin were drawn from the pathology files of the Institute of Pathology (University Wuerzburg).

Tape stripping of skin was performed with ethanol-washed tails from WT and *Nfat5^-/-^* mice according to Ref (23). using corneofix tapes (Courage + Khazaka, Cologne).

### Statistical Analysis

Statistical analysis was performed by using GraphPad Prism 6.0 (GraphPad Software, San Diego, USA). Data was presented as mean and error bars in figures represent ± SEM. Unpaired t-tests was used to evaluate the statistical differences. The values ≤0.05 were considered significant. ***p<0.0001, **p<0.001 and *p<0.05.

## Results

### NFAT5 Is Expressed in Basal and Suprabasal KCs of Epidermis

Ca^++^ play an important role in epidermal differentiation (10). Therefore, one may speculate that the Ca^++^/calcineurin/NFATc signaling cascade, which plays a central role in lymphocytes, might also control epidermal differentiation. However, when we investigated the expression of NFATc1 and NFATc2, the two most abundant NFAT factors in lymphocytes, in immunoblots, we observed a relatively poor NFATc expression in KCs, compared to lymphocytes. By contrast, the osmo-sensitive factor NFAT5, a distant relative of genuine NFATc factors (13), was expressed at a similar level in KCs and lymphocytes (**Supplementary Figures 1A, B**). This observation prompted us to study the role of NFAT5 in KCs.

Co-stainings of skin sections from adult humans with antibodies (Abs) raised against NFAT5 and markers of basal KC, as keratin 14 (K14) (6), or of suprabasal KC, as filaggrin (24), revealed NFAT5 expression throughout the epidermis. In numerous basal KCs, NFAT5 expression appeared to be restricted to the cytoplasm, whereas in suprabasal KCs we detected NFAT5 expression predominantly – albeit not exclusively – in the nuclei of KCs (**Supplementary Figures 1C, D**). Under pathophysiological and experimental *in vitro* conditions, such as in epidermis from Ichthyosis patients and of sun-exposed persons, NFAT5 was predominantly expressed in suprabasal KCs (**Supplementary Figures 1D, E**).

We also stained NFAT5 in cytospins of cultured murine KCs. By using Abs raised against a C-terminal NFAT5 epitope (ab3446) or an Ab directed against an internal region of human NFAT5 (ab110995) we detected NFAT5 both in cytosol and nuclei of KCs. While the ab3446 Ab stained predominantly the nuclei of cultured KC, the ab110995 Ab stained their cytosol indicating the expression of NFAT5 in both cellular compartments of basal KCs in culture (**Supplementary Figures 2A, B**). This suggest a complex expression of NFAT5 in multiple isoforms in KCs, as described previously for other cells (25).

**Figure 1:**
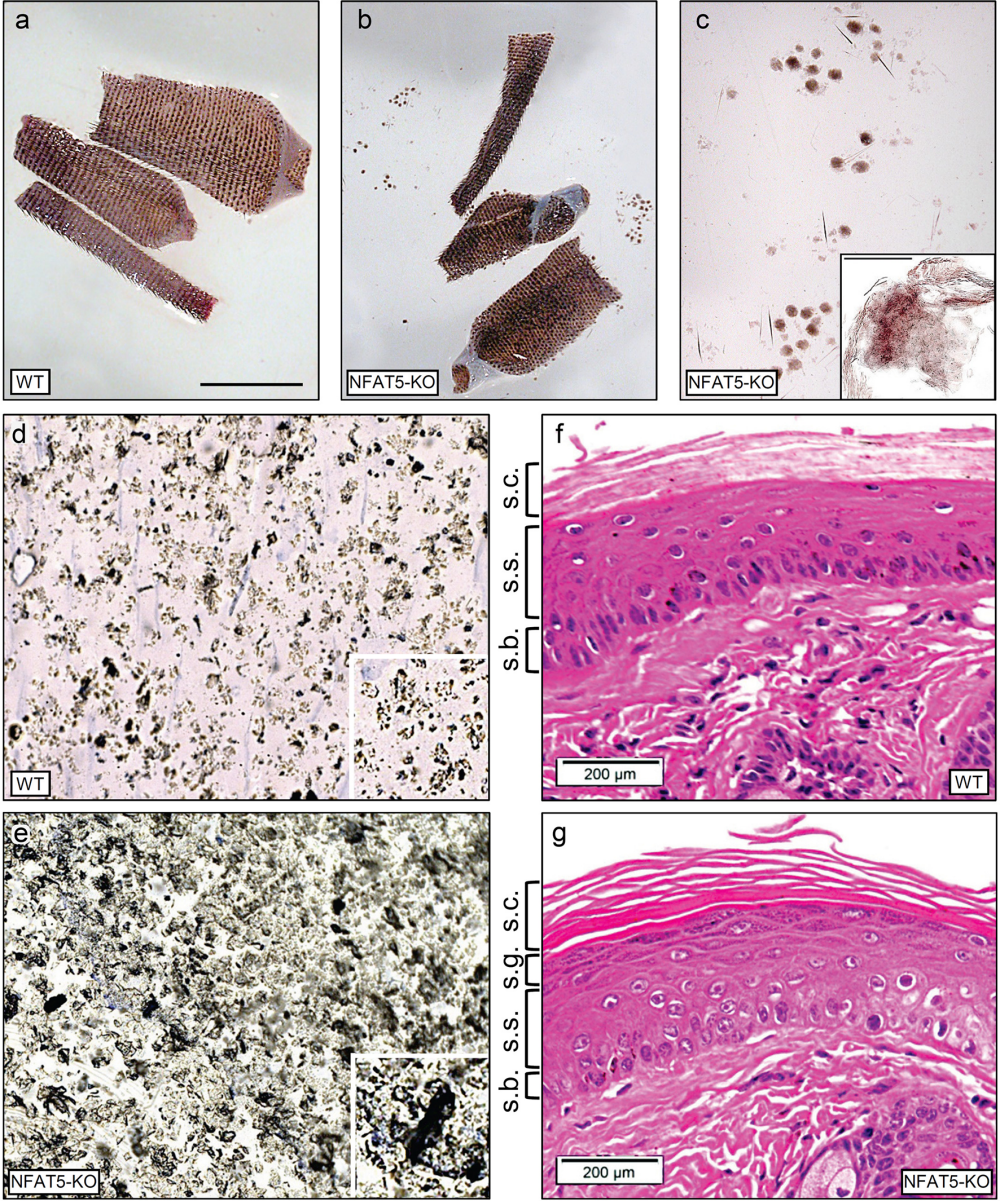
NFAT5 ablation changes the integrity and morphology of murine epidermis. **(A, B)** Preparations of skin from 129sv WT **(A)** and 129sv *Nfat5^-/-^* mice **(B, C)**. In **(C)**, skin sheets released from tail skin of *Nfat5^-/-^* mice are presented at larger magnification. Bar= 100 μM. **(D, E)** Tape strips from the tails of WT **(D)** and *Nfat5^-/-^* mice **(E)**. One representative strip of more than ten is shown. **(F, G)** Representative H&E stains of skin sections from tails of WT **(F)** and *Nfat5^-/-^* mice **(G)**. s.c., *stratum corneum*; s.g., *stratum granulosum*; s.s., *stratum spinosum*; s.b., *stratum basale*.

Ablation of NFAT5 in mice did not lead to gross changes of skin morphology (15, 26). However, when we prepared the skin and KCs from tails of *Nfat5^-/-^* mice, we detected the release of large skin sheets into the preparation medium that we never observed during the preparation of WT tail skin (**Figures 1A–C**). These sheets consisted of corneocytes and suprabasal KCs (see insert in **Figure 1C**). The fragile skin from *Nfat5^-/-^* mice was also observed in tape stripping assays (23) using tails from WT and *Nfat5^-/-^* mice (**Figures 1D, E**). The first tape strips from the skin of *Nfat5^-/-^* mouse tails revealed a tight layer of corneocytes packed by skin scales which differed in density and their large size from those of WT mice. While the skin of *Nfat5^-/-^* mice formed frequently a patchwork of large, released scales, the WT corneocytes showed a loose assembly of single scales (see inserts in **Figures 1D, E**). Sections through the tail skin of *Nfat5^-/-^* mice revealed a more fragile *stratum corneum* than in WT skin (compare **Figures 1F** with **1G**), and in numerous sections of tails from *Nfat5^-/-^* mice the *stratum corneum* was completely lost (**Supplementary Figure 3**).

### NFAT5 Affects Protein Secretion by KCs

The cornification of KCs and desquamation of corneocytes is controlled by numerous proteases that are secreted by KCs (8). These proteases cleave the proteins of extracellular desmosomes to allow shedding of corneocytes. In order to elucidate whether NFAT5 affects the secretion of murine KCs we isolated KCs from tails of WT and *Nfat5^-/-^* mice and cultured them for 2-4 weeks in serum-free SFP KC medium (Gibco) containing 0.06 mM Ca^++^. Under these low Ca^++^ conditions, the basal KCs generated a layer of cobblestone-like KCs within a few days. However, upon culture for 3 weeks and longer the confluent primary KCs started to enlarge their size and formed cornification-like envelopes in the lawn of KCs (**Supplementary Figures 4A, B**).

**Figure 2:**
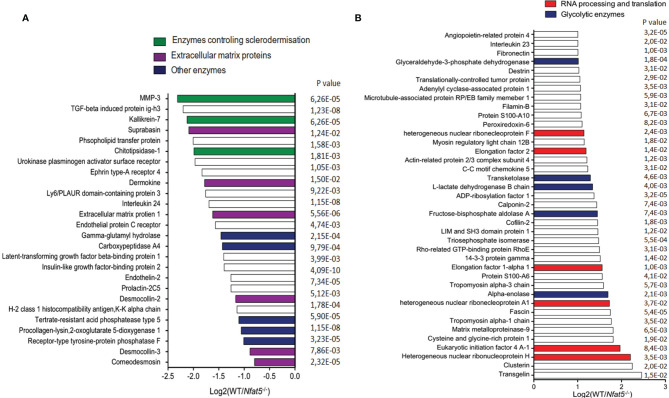
Proteins that were secreted by isolated tail KCs within 24 h of culture. **(A)** Proteins that were 2-fold and more secreted by 129sv *Nfat5^-/-^* than 129sv WT KCs. **(B)** Proteins that were at least 2-fold less secreted by *Nfat5^-/-^* than WT KCs. Data of two independent experiments each analyzed in triplicates by LC-MS were compiled.

Using a novel metabolic labelling technique, we determined the secretome of WT and *Nfat5^-/-^* KCs. To this end, KC proteins were labelled with azidohomoalanine (AHA) for 20 h, followed by avidin-based affinity purification of secreted proteins, on-resin trypsinisation and mass spectroscopy (21). In those assays, we detected approximately 300 proteins that were secreted by WT and *Nfat5^-/-^* KCs within 24 h. Among the 24 proteins whose secretion was increased twofold and more by *Nfat5^-/-^* cells as compared to WT KCs we detected four extracellular matrix proteins and three enzymes (**Figure 2A**). The three enzymes matrix metallopeptidase 3 (Mmp3, encoded by the *Mmp3* gene; also known as stromelysin-1), kallikrein 7 (*Klk7*), and chitotriosidase 1 (*Chit1*) that showed a strong increase in secretion by *Nfat5^-/-^* compared to WT KCs are known to cleave and process extracellular matrix proteins and to control the sclerodermisation of epithelia (27–29). Three of the matrix proteins, suprabasin (*Sbsn*), dermokine (*Dmkn*) and desmocollin 2 (*Dsc2*) are known to be involved in the terminal differentiation of corneocytes, and two further cornification proteins, desmocollin 3 (*Dsc3*) and corneodesmin (*Cdsn*) (30–32), were also stronger secreted by *Nfat5^-/-^* than WT KCs (**Figure 2A**).

Within the pattern of 39 proteins whose secretion by *Nfat5^-/-^* KCs was diminished twofold and more, neither any cornification protein nor any protease, except Mmp9, was detected. Instead, *Nfat5^-/-^* KCs showed a decrease in several proteins that control RNA processing and translation, and enzymes that control cellular metabolism, as the glycolytic enzymes alpha enolase 1 (*Eno1*), fructose-biphosphate aldolase A (*Aldoa*), L-lactate dehydrogenase B (*Ldhb*), transketolase (*Tkt*) and glyceraldehyde-3-phosphate dehydrogenase (*Gapdh*) (**Figure 2B**). However, when we investigated the incorporation of glucose into cultured KCs using the fluorescent glucose analog 2-[N-(7-nitrobenz-2-oxa-1,3-diazol-4-yl) amino]-2deoxy-D-glucose (2-NBDG) we observed a 2-3-fold increase in 2-NBDG uptake into *Nfat5^−/−^*, compared to WT KCs (**Supplementary Figure 5**). This suggests a suppressive influence of NFAT5 on the metabolism of basal KCs.

### NFAT5 Orchestrates KC Differentiation

To elucidate whether and how NFAT5 affects gene transcription in KCs, we used NGS assays to compare the transcriptomes of WT and *Nfat5^−/−^* KCs prepared from tails of adult mice. Due to the large variations that we observed in initial assays between various preparations of tail KCs, we first compared the transcriptomes of the same batches of KCs from WT C57BL/6J mice that were cultured either for one or for three weeks. To our surprise, we detected fundamental differences in the expression levels of numerous genes at these time points (**Supplementary Figure 6A**). The marked differences in gene expression profiles of 129/sv WT versus *Nfat5^−/−^* KCs (see **Supplementary Figure 6B**) might reflect the rapid differentiation events that change gene expression between basal and suprabasal KCs in murine skin during differentiation. Therefore, all transcriptomes that we obtained reflect a snapshot of a restricted life period of basal KCs. To keep this in mind, in the following we present and discuss the transcriptome results of KCs from two129/sv WT and *Nfat5^−/−^* sibling mice.

**Figure 3:**
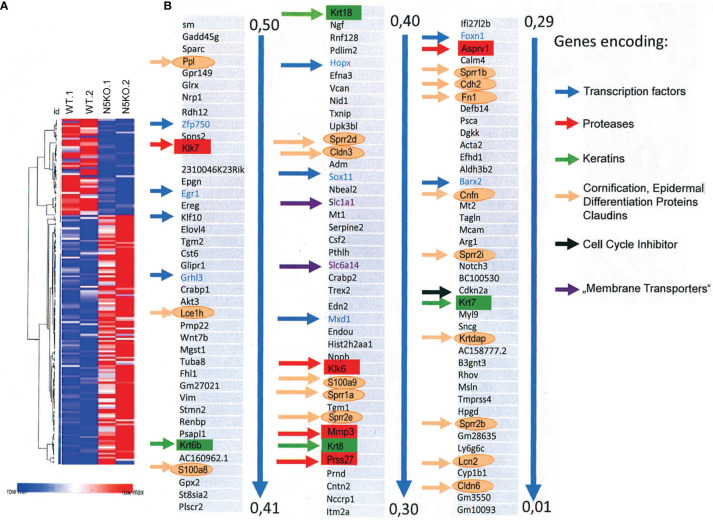
Ablation of NFAT5 changed gene expression in cultivated KCs. **(A)** Heat map of 168 genes that were changed at least twofold in 129sv *Nfat5^-/-^* compared to 129sv WT KCs. **(B)** Compilation of 121 genes that were expressed in 2-fold more copies in *Nfat5^-/-^* KCs. Nine genes encoding TFs are shown in blue and indicated by blue arrows. Five protease genes are boxed and shown in red, four keratin genes are shown in green boxes and by green arrows. The gene coding for the cell cycle inhibitor *Cdkn2a* is shown in black, genes encoding membrane transporters are shown violet, and the numerous genes encoding Epidermal Differentiation Complex (EDC) proteins, claudins and other ‘cornification` proteins are encircled and presented in orange.

Among the 121 genes that were expressed in two and more RPKMs (Reads Per Kilobase Million, total reads in a sample divided by 1 mill.) in *Nfat5^-/-^* compared to WT KCs we detected nine genes encoding TFs (see blue arrows in **Figure 3**). Four of the TF genes, *Mxd1* (encoding the Myc partner Mad), *Hopx, Grhl3* and *Klf10* are known to control the proliferation of KCs (33–36), *Barx2* and *Hopx* regulate hair follicle differentiation (37, 38) while *Foxn1*, *Sox11* and *Egr1* are expressed during wound repair (39–41) and *Egr1* and *Zfp750* in psoriatic lesions (42, 43) (**Supplementary Table 2**). Only one TF gene, the *Irf9* gene, which is highly expressed in psoriatic lesions (44), appeared in the list of the 47 genes that were expressed 2-fold stronger in *Nfat5^-/-^* than WT KCs **(** **Supplementary Figure 7** and **Supplementary Table 2** **)**.

**Figure 4:**
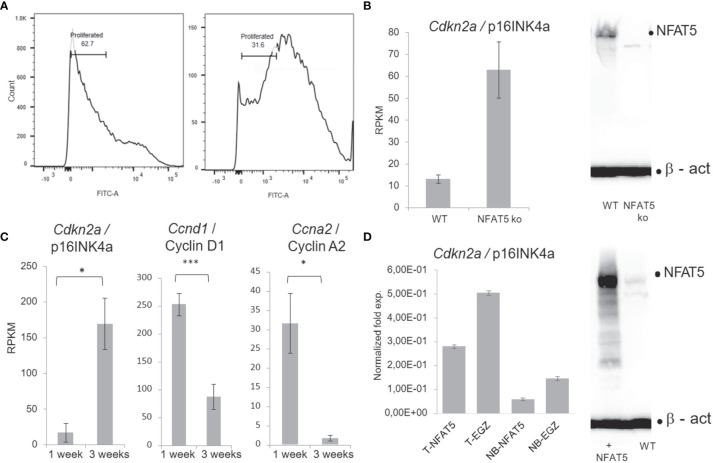
NFAT5 supports the proliferation of KCs. **(A)** Measurement of KC proliferation cultivated for one week (left) or three weeks (right) in CFSE assays. **(B)** Increase of p16^INK4a^ RNA levels in KCs upon NFAT5 ablation. The western blot (right) confirms the absence of NFAT5 expression in KCs isolated from *Nfat5^-/-^* mice. **(C)** Expression of cell cycle inhibitor p16^INK4a^ and of cyclins D1 and A2 in KCs cultivated for one or three weeks. Next-generation-sequencing (NGS) data of four independent batches of KCs cultured for 1 or 3 weeks are shown. **(D)** Decrease in p16^INK4a^ RNA levels in KCs isolated from the tails of adult (T) or from newborn mice (NB). The western blot illustrates increased NFAT5 levels in tail KCs that have been transduced with a retrovirus expressing NFAT5. The KCs in **(C, D)** were cultivated for 1 week. (***p<0.0001, **p<0.001 and *p<0.05).

The differentiation of basal towards suprabasal KCs is closely associated with a reduction in cell proliferation. A diminished proliferation was observed *in vitro* upon prolonged culture of primary KCs at the cellular (**Figure 4A**) and molecular levels. KCs cultured for one week divided rapidly and expressed relative low levels of the cell cycle inhibitors *Cdkn1a*/p21^WAF^, *Cdkn2a*/p16^INK4a^ and *Cdkn2b*/p15^INK4b^ whereas after 3 weeks and more they slowed down in proliferation while expressing up to 30-fold higher levels of cell cycle inhibitors. On the contrary, the expression of cyclins *Ccnd1*/cyclin D1, *Ccna1*/cyclin A2, *Ccnb1*/cyclin B1 and B2, and of cyclin-dependent kinase 1 (Cdk1) was much stronger in KCs cultured for 1 week than in cells cultured for 3 weeks (**Figure 4** and **Supplementary Figure 8**). The drop in KC proliferation was also reflected in a decrease of components of RNA polymerase I whose expression reflects cell cycle progression (45) (**Supplementary Figure 8D**). However, among the numerous genes that control KC proliferation the *Cdkn2a* gene seems to be the only gene whose expression is controlled directly by NFAT5. In *Nfat5^−/−^* KCs we detected a 4- to 6-fold increase in *Cdkn2a* transcripts whereas in KCs transduced with retroviruses expressing NFAT5 a marked reduction in proliferation and levels of *Cdkn2a* transcripts was observed (**Figures 4C, D**).

Numerous genes encoding cornification, tight junction and adhesion proteins belong to the group of genes that were stronger transcribed in *Nfat5^-/-^* than in WT KCs. Among those are ten genes that code for proteins of the Epidermal Differentiation Complex (EDC). These are seven small proline-rich proteins (Sprr1a, Sprr1b, Sprr2b, Sppr2d, Sppr2e, Sprr2h and Sprr2i), two S100 family proteins (S100a8 and S100a9), and the late cornified envelope protein Lce1h. Further genes that are suppressed by NFAT5 encode lipocalin 2 (*Lcn2*), the tight junction proteins claudins 3 and 6 (*Cldn3* and *6*), cornefilin (*Cnfn*), cadherin 2 (*Cdh2*), fibronectin 1 (*Fn1*), the KC differentiation-associated protein (*Krtdap*), and the *Tgm1* and *Tgm2* genes (**Figure 3** and **Supplementary Figure 9**).

Apart from the *S100a7a* gene encoding the S100 calcium binding protein A7A that is highly expressed in psoriatic lesions (46), none of these ‘KC-differentiation’ genes were found in the list of genes that were decreased in expression in *Nfat5^-/-^* KCs (**Supplementary Figure 7**). The majority of ‘differentiation’ genes did not increase but decrease upon prolonged culture of KCs for 3 weeks. Exceptions are the *Fn1, Tgm2* and *Lcn2* genes whose expression increased upon prolonged culture of tail KCs. On most of these genes, over-expressing NFAT5 upon transduction of NB and tail KCs exerted only a moderate effect on expression levels suggesting that they are no direct targets of NFAT5 (**Supplementary Figure 9**).

### NFAT5 Modulates Keratin Expression in Epidermal KCs

Within the transcriptome of basal KCs, we detected a large number of transcripts for keratin K5/K14 heterodimers, the most abundant keratins of basal KCs (47). The K14 transcripts decreased slightly upon culture for 3 weeks, whereas a marked 2-3 fold decrease in K5 RNA levels was detected. In contrast, the transcripts of keratins 6a, K7, K8 and K18 increased 2 to 6-fold in KCs cultured for 3 weeks as compared to KCs cultured for only one week (**Supplementary Figure 10**). The transcripts of K5/14 heterodimers were at the same high level in WT and *Nfat5^-/-^* KCs, and they remained unaffected by NFAT5 expression in isolated tail KCs while they were slightly repressed in NB KCs. By contrast, the expression of K7, K8 and K18 keratins increased 2 to 6-fold in *Nfat5^-/-^* KCs, and upon NFAT5 over-expression a slight decrease in K8 and K18 RNA levels was observed (**Supplementary Figure 10**). It is noteworthy that no transcripts were detected for the keratins K1 and K10, the markers of suprabasal KCs.

### Coordinated Expression of NFAT5 and Mmp3 in Fetal and Adult Skin

**Figure 5:**
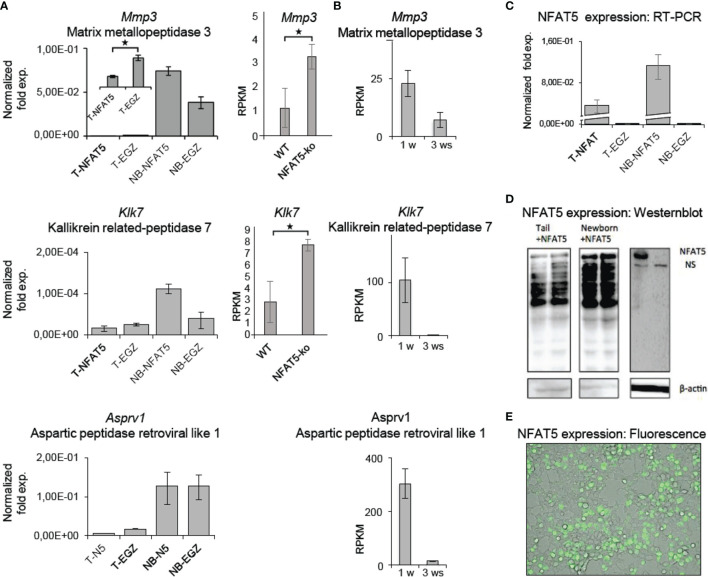
NFAT5 affects the expression of *Mmp3, Klk5* and *Asprv1* protease genes in KCs. **(A)** Left panels, results of qRT-PCR assays showing the effect of NFAT5 on the expression of *Mmp3, Klk7* and *Asprv1* genes upon transduction of KCs obtained from the tails of adult ice (T) or from the skin of newborn (NB) mice. Right panels, effect of NFAT5 ablation on *Mmp3* and *Klk7* expression. Results of 3 independent NGS assays are shown. **(B)** Expression of protease genes in KCs cultured for 1 or 3 weeks (ws). NGS data of three independent batches of KCs cultured for 1 or 3 ws are shown. **(C–E)** Detection of NFAT5 expression upon transduction of KCs. Cultured KCs were transduced with retroviruses expressing NFAT5, or with an ‘empty’ EGZ virus. **(C)** Detection of human NFAT5 RNA by RT-PCR assays in tail (T) and NB KCs upon transduction. **(D)** Western blots showing the expression of NFAT5 upon transduction of KCs from newborn and tails of adult mice. **(E)** Fluorescence microscopy of NFAT5-GFP expression in transduced adult KCs. (*p<0.05).

Among the 121 genes that were 2-fold stronger expressed in *Nfat5^-/-^* than WT KCs we detected five genes that encode matrix proteases (**Figure 3**). In line with the protein secretion data, these were the *Mmp3* and *Klk7*, and, in addition, the *Klk6*, *Asprv1* and *Prss27* genes. In qRT-PCR assays, we observed a strong increase in *Mmp3, Klk7* and *Asprv1* expression in KCs from NB mice as compared to adult KCs, and a decrease in tail KCs that were maintained for 3 weeks *in vitro* (**Figure 5**). The *Mmp3* gene was 30- to 50-fold stronger expressed in NB KCs as in adult KCs, and over-expressing NFAT5 in NB KCs led to a moderate increase in RNA levels. In contrast, the poor expression of *Mmp3* gene in adult KCs was suppressed 2 to 3-fold upon NFAT5 over-expression. A similar expression was observed for the *Klk7* gene: a stronger expression in NB than in adult KCs, a stimulatory effect of NFAT5 on *Klk7* expression in NB but a slight repression in adult KCs. Moreover, expression of the *Klk7* gene ceased in adult KCs upon prolonged incubation for 3 weeks. In 3 independent transcriptome NGS assays the RNA levels of *Mmp3* and *Klk7* genes were 2-3 fold higher in *Nfat5^-/-^* than WT tail KCs (**Figure 5**).

**Figure 6:**
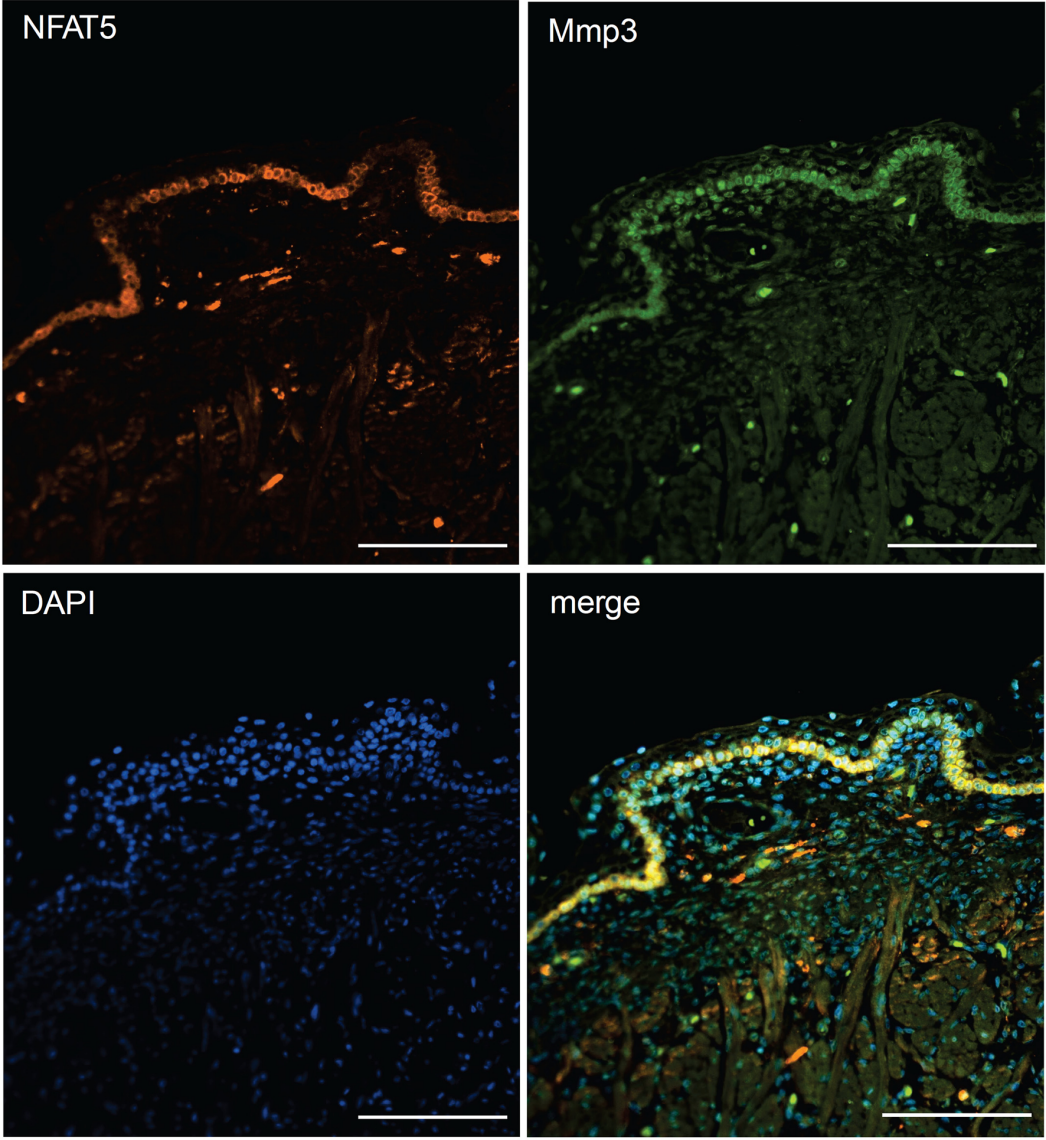
Immunofluorescence of sections through the skin of a human fetus. Sections were stained with Abs against NFAT5 (ab110995, Abcam) and Mmp3 (AF513, Novus Biologicals) and counterstained with DAPI as indicated. Length of the bars: 100 μm.

**Figure 7:**
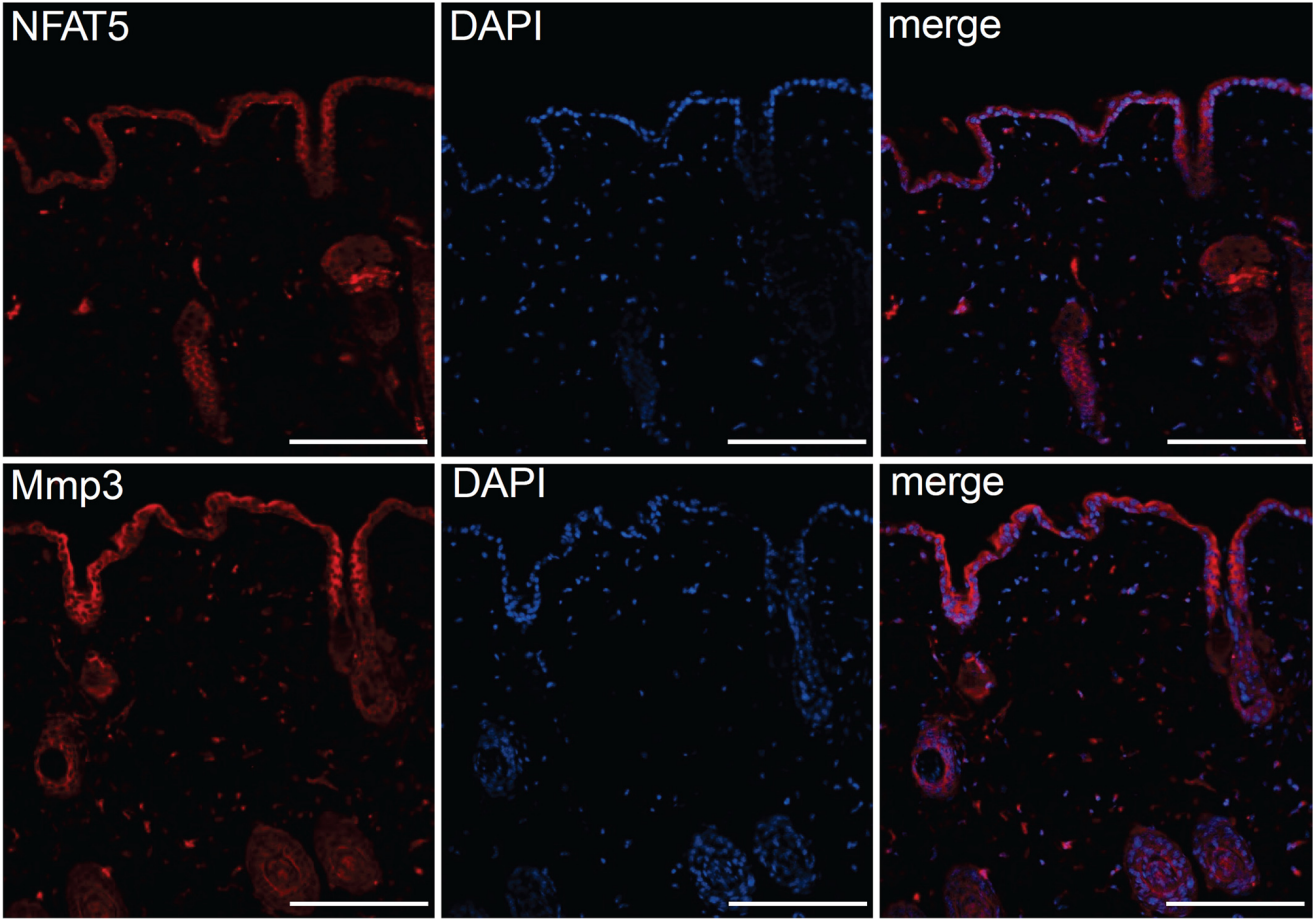
Immunofluorescence of serial sections through the back skin of adult mice. Sections were stained with Abs against NFAT5 (ab110995, Abcam) and Mmp3 (ab53015 (Abcam) and counterstained with DAPI as indicated. Length of the bars: 100 μm.

**Figure 8:**
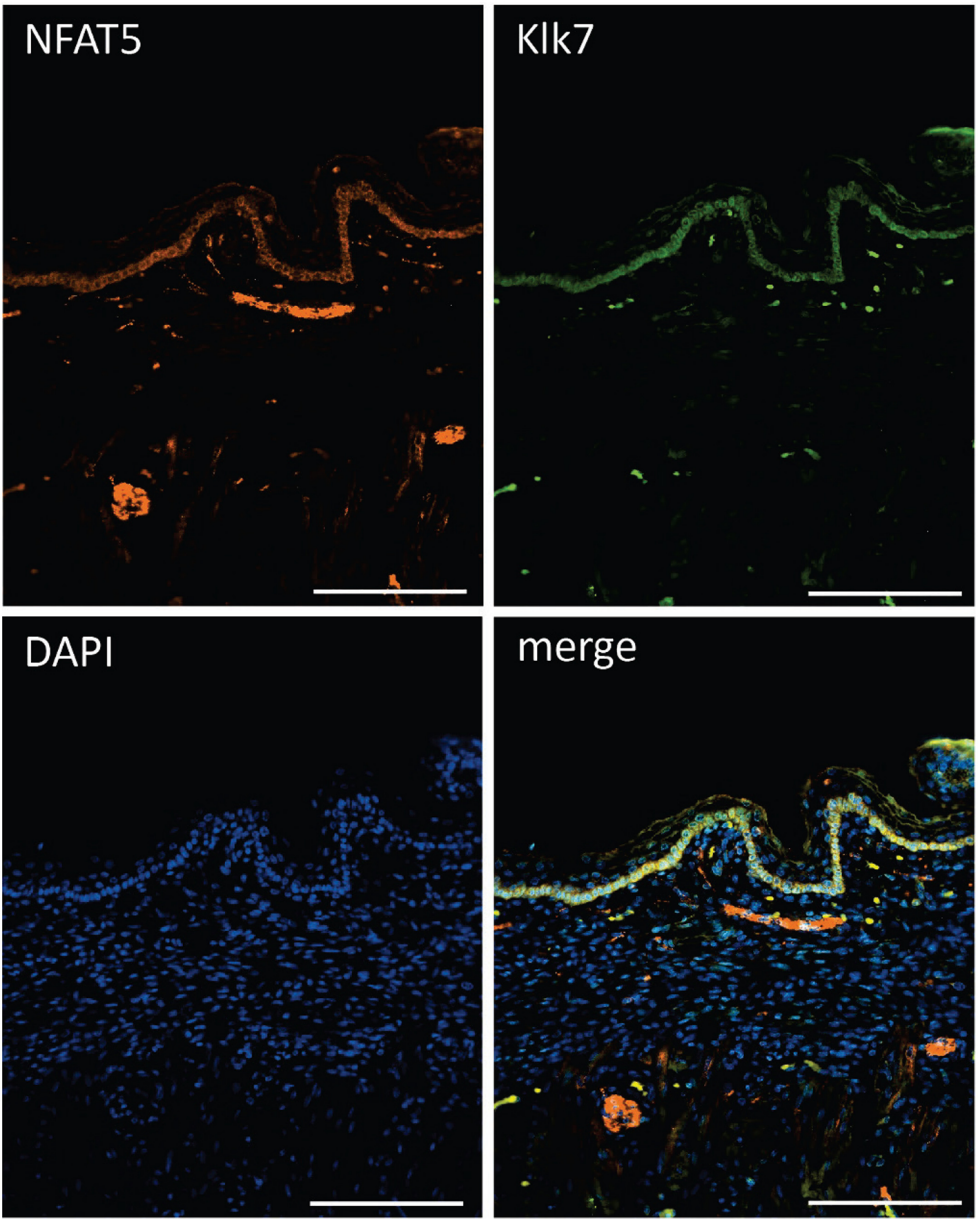
Immunofluorescence of sections through the skin of a human fetus. Sections were stained with Abs against NFAT5 (ab110995, Abcam) and Klk7 (Biotechn/R+D Systems, No.: AF2624) and counterstained with DAPI as indicated. Length of the bars: 100 μm.

The striking variations in expression of matrix proteases prompted us to investigate whether and, if yes, where and when NFAT5, Mmp3 and Klk7 are co-expressed at various developmental stages. Therefore, we (co-) stained skin sections of adult, newborn and fetal mice with Abs specific for NFAT5, Mmp3 or Klk7. Since the anti-murine Mmp3 Abs did not allow co-stains of murine skin, we co-stained skin sections of human fetuses that revealed a distinct co-expression of NFAT5 and Mmp3 in KCs of *stratum basale*. In the cytosol of almost all KCs of the basal epidermal layer a strong NFAT5 and Mmp3 co-expression was detected, and a number of KCs showed also the nuclear occurrence of NFAT5 (and Mmp3) (**Figure 6**). A similar staining pattern was detected in sections of adult murine skin in which the NFAT5 and Mmp3 specific Abs stained predominantly basal KCs (**Figure 7** and **Supplementary Figure 11**). On the same hand, an Ab specific for Klk7 stained also the layer of basal KCs in sections of human fetuses in which NFAT5 is expressed in cytosol (**Figure 8**).

In striking contrast, we did not detect any NFAT5 expression in KCs of the *stratum basale* from NB mice while a strong and broad Mmp3 staining was apparent (**Supplementary Figure 11**). A similar staining pattern was observed in stainings of NB epidermis with an Ab specific for Klk7: while the Klk7 Ab stained broadly the epidermis of NB mice, no NFAT5 staining was detected. By contrast, the basal KC layer of epidermis from adult mice was co-stained by both Klk7 and NFAT5 (**Supplementary Figure 12**). These data suggest that in KCs from *stratum basale* of embryonic and adult mice – but not of NB mice - the co-expression of NFAT5 with Mmp3 and Klk 7 keeps in check the expression of matrix proteases in epidermis.

## Discussion

NFAT5 was originally described as an osmosensitive TF that affects the activity of T cells, macrophages, and other cells in the skin. Thereby, increasing Na^+^ concentrations support the antimicrobial skin barrier function (3, 48). However, to our surprise we did not detect any role for NFAT5 in controlling Na^+^ transport by KCs, nor an effect on epidermal *Nos2* expression, a known NFAT5 target gene in macrophages (49). Therefore, similar to NFAT5’s role in neutrophils where glucocorticoid hormones control NFAT5 expression and activity (4), in epidermal KCs NFAT5 exerts functions other than salt uptake and secretion.

The data of our study indicate that in KCs of inter-follicular epidermis NFAT5 controls the terminal differentiation of KCs to corneocytes. Although we investigated predominantly the role of NFAT5 in cultured basal KCs, the molecular mechanisms that control the differentiation of those cells to suprabasal KCs are initiated by a hierarchy of events that lead, finally, to the aberrant cornification of skin in *Nfat5^-/-^* mice. This is reflected by the skin phenotype of *Nfat5^-/-^* mice and by numerous changes in gene expression and protein secretion that we observed in cultured basal KCs (i.e., KCs that resemble those of the basal epidermal layer, in the *stratum basale*).

At the molecular level, NFAT5 (i) supports the proliferation and (ii) represses the differentiation of basal KCs. This is documented best by the massive increase of cell cycle inhibitor p16^INK4a^ upon ablation of NFAT5, by the increase in expression and secretion of matrix proteases and of numerous members of the epidermal cornification complex in *Nfat5^-/-^* KCs. Moreover, NFAT5 controls the expression of a set of TFs, which are closely associated with proliferation and differentiation of KCs.

NFAT5 represses the expression of nine TF genes. One of them, MAD1 that is encoded by the *Mxd1* gene, is closely associated with the differentiation of KCs (33). MAD1 (MAX dimerization protein 1), a transcriptional repressor, is a dimerization partner of Myc and highly expressed in suprabasal KCs. Sox11 and its relative, Sox4, have been described to support an embryonic gene expression program that becomes activated during wound healing (40). A function in skin regeneration was also described for the expression of *Foxn1* (39), the ‘nude locus’ encoding Whn (50), that is particularly expressed in the *stratum spinosum*. While *Foxn1* appears to initiate KC differentiation by controlling more than 50 genes, it is unable to induce final differentiation steps (51, 52). An important function in wound healing and repair processes has been ascribed to *Grhl3*, which, in addition, controls the skin barrier function (35). *Hopx* expression, on the other hand, inhibits terminal differentiation of human KCs (34).

It remains to be shown by which signals the expression and function of NFAT5 is controlled in epidermal KCs. One upstream mediator is probably Blimp-1, a transcriptional repressor, whose epidermal ablation in mice resulted in defects in differentiation of KCs from the *stratum granulosum* to the *stratum corneum* and, thereby, to skin barrier defects. Among the 250 genes that were dysregulated in Blimp-1-deficient epidermis, *Nfat5* was identified as a direct target of Blimp-1. However, the genes that we described here as NFAT5 targets in KCs were not detected as (direct or indirect) targets of Blimp-1 (53) thus questioning the involvement of NFAT5 in Blimp-1-mediated repression of KC genes.

Among the genes that are stronger transcribed in *Nfat5^-/-^* than WT KCs are numerous genes that control late cornification events. These are seven *Sprr* genes coding for small proline-rich region (SPRR) proteins, the *Lcn2*, the *Lce1h* gene encoding a member of late cornified envelope (LCE) proteins, *Cnfn*, encoding cornifelin, the *Fn1* gene encoding the extracellular matrix protein fibronectin 1, two genes encoding the tight junctions proteins claudin 3 and 7, and genes encoding S100 proteins. In addition, the *Tgm1* and *Tgm2* genes are further ‘cornification genes’ that are stronger expressed in *Nfat5^-/-^* than in WT KCs. They encode the transglutaminases 1 and 2, which cross-link cornification proteins by catalysing N6-(γ-glutamyl) lysine isopeptide bonds. Albeit both genes are repressed by NFAT5, their expression differs markedly between KCs that were kept in culture for either 1 or 3 weeks, respectively. While the *Tgm1* gene is predominantly expressed in freshly prepared KCs, *Tgm2* is much stronger expressed after 3 rather than 1 week of culture. Tgm1 defects have been associated with aberrant cornification and ichthyosis (54), whereas Tgm2 acts in many tissues and stabilizes extracellular matrices. Defects of the latter are closely linked with various diseases in humans (55).

Our KC secretion and transcriptome studies from adult *Nfat5^-/-^* mice revealed the peptidases Klk7 and Mmp3 as prominent extracellular matrix proteases whose synthesis is suppressed by NFAT5. Both proteases are known to cleave a multitude of extracellular matrix proteins, including corneodesmosomes (56, 57), and, thereby, control the shedding of corneocytes. The expression of both genes is controlled at the transcriptional level (58, 59), and it is likely that NFAT5 keeps in check their expression in adult (and embryonal) KCs. In contrast, we observed a strong, up to 50-fold increase in expression of both proteases in KCs from NB mice and a very low, if any NFAT5 expression in NB KCs. This suggests that NFAT5 does not repress Mmp3 expression in the epidermis of NB mice at the time point of birth when the skin barrier function is established. One may speculate that for the re-organization of the skin barrier which at birth has to immediately adapt from an amniotic fluid environment to air contact high levels of matrix proteases are necessary which is made possible by low expression levels of epidermal NFAT5.

## Data Availability Statement

The datasets presented in this study can be found in online repositories. The names of the repository/repositories and accession number(s) can be found below: Sequencing data - NCBI GEO, accession no: GSE184180; Mass spectrometry data - PRIDE database, accession no: PXD028675.

## Ethics Statement

Ethical review and approval was not required for the study on human participants in accordance with the local legislation and institutional requirements. Written informed consent for participation was not required for this study in accordance with the national legislation and the institutional requirements. Animal experiments were performed according to project licenses (Nr. 55.2-2531.01-80/10), which are approved and controlled by the Regierung von Unterfranken, Würzburg. Written informed consent was not obtained from the individual(s) for the publication of any potentially identifiable images or data included in this article.

## Author Contributions

ES: led the investigation and wrote the manuscript. KMuh, DX, MA, TR, and AA: performed experiments. MS and ST: performed and evaluated mass spectrometric analyses of secretion assays. SK-H, MK, and TB: NGS assays. KMur and AK: Immunofluorescence and -chemistry stains. MG: supported the preparation of the manuscript. All authors contributed to the article and approved the submitted version.

## Funding

This work was supported by the Deutsche Forschungsgemeinschaft (DFG; SE469/24-1/27-1; GO811/5-1/6-1; KE1343/2-1/3-1, TR/SFB156, TPB11N to TB, and SFB1292, TPZ01 to ST), the Wilhelm-Sander-Stiftung (to ES and S K-H), and the IZKF Würzburg (Project A-371 to KM, AK and ES and AdvCSP-2 to AK).

## Conflict of Interest

The authors declare that the research was conducted in the absence of any commercial or financial relationships that could be construed as a potential conflict of interest.

## Publisher’s Note

All claims expressed in this article are solely those of the authors and do not necessarily represent those of their affiliated organizations, or those of the publisher, the editors and the reviewers. Any product that may be evaluated in this article, or claim that may be made by its manufacturer, is not guaranteed or endorsed by the publisher.

## Supplementary Material

Supplementary Figure 1 NFAT5 and NFATc1 expression in epidermis of inter-follicular skin of mice and human. **(a)** Parallel Western blots with the same batches of whole cell protein extracts from primary murine KCs, compared to whole cell extracts from murine CD4^+^T cells. Upper blot, detection of NFATc1 expression by incubation with the NFATc1-specific mAb 7A6. Lane 1, murine CD4^+^ T cells activated by T+I for 24 h; lanes 2-5, KCs incubated for 3d in serum-free SFM KC medium (Gibco) containing 0.06 mM Ca^++^ without (lane 2) and with ionomycin (0.5 μM; lane 3), cyclosporine A (100 ng/ml; lane 4) or imiquimod (1 μg/ml; lane 5). In lanes 6-13, KCs were incubated in SFM medium containing 0.2 mM CaCl_2_ without (lane 6) and with ionomycin (lane 7), with cyclosporine A (lane 8), imiquimod (lane 9), ionomyin+cyclosporine A (lane 10) or ionomycin+imiquimod (lane 11). In lane 12 and 13, KCs were incubated in S-MEM medium containing 0.2 mM CaCl_2_ without (lane 12) or with ionomycin (lane 13). Lower blot, NFAT5 expression detected by pAb PAI-023 (Affinity Bio Reagents). For the lanes see **(a)**. **(b)** Immuno-histochemical staining of a section through human skin stained with the NFATc1-specific mAb 7A6. **(c)** Co-staining of sections through human back skin with Abs specific for NFAT5 (ab3446) and cytokeratin 14 (sc-53253). **(d)** Co-staining of human back skin with Abs for NFAT5 (ab3446) and filaggrin (sc-66192). **(e)** Staining of human sun-exposed face skin with an NFAT5-specific Ab (sc-398171). An enlargement of framed part of **(e)** is shown in **(é)**. Length of the bars: 50 μm.

Supplementary Figure 2 Localization of NFAT5 in murine tail KCs upon culture for 1 week. Upon cytospin, the KCs were stained with the NFAT5-specific Abs ab3446 **(a)** or ab110995 **(b)**.

Supplementary Figure 3 Representative H&E stains of sections through the skin from tails of WT129/sv **(a)** and *Nfat5^-/-^* 129/sv mice **(b)**. Note the missing corneocyte layer in skin from *Nfat5^-/-^* mice.

Supplementary Figure 4 Primary KCs from tails of WT C57/Bl6 mice cultured for one week **(a)** or three weeks **(b)** *in vitro*.

Supplementary Figure 5 Increased uptake of the fluorescent glucose analog 2-NBDG (2-[N-(7-nitrobenz-2-oxa-1,3-diazol-4-yl) amino]-2deoxy-D-glucose) into NFAT5-deficient 129/sv KCs, as compared to WT 129/sv KCs. **(a)** Flow cytometry of FITC-labeled 2-NBDG, and **(b)** quantification of 2-NBDG uptake.

Supplementary Figure 6 Heat maps of more than 8400 genes that were expressed in 3 and more RPKMs in basal murine KCs. **(a)** Transcriptomes of KCs from WT C57BL/6 mice cultured for one or three weeks *in vitro*. Data from four independent experiments were compiled with cells being cultured for either 1 or 3 weeks. **(b)** Heat maps of transcriptomes from KCs of 129/sv mice. The two WT and *Nfat5^-/-^* KC cultures were maintained *in vitro* for 1 week.

Supplementary Figure 7 Compilation of genes that were 2fold weaker expressed in *Nfat5^-/-^* than WT 129/sv KCs. **(a)** Heat map of 168 genes that were changed at least 2fold in expression between WT and *Nfat5^-/-^* KCs. **(b)** Compilation of 47 genes that were expressed in 2fold less copies in *Nfat5^-/-^* KCs. The gene encoding the TF Irf9 is marked by a blue arrow. Five genes encoding membrane transporters are highlighted by violet arrows. Genes coding for cytokine receptors and Tnf-signaling molecules are indicated by green arrows, and the laminin gene *Lamb3* and the gene encoding *S1007a*, a “cornification protein”, are indicated by a grey or red arrows, respectively.

Supplementary Figure 8 Extended culture of WT C57BL/6 KCs affects the expression of genes that control cell cycle. **(a)** Genes encoding cell cycle inhibitors. **(b)** Cyclin genes. **(c)** Cyclin-dependent kinase *Cdk1* gene. **(d)** Genes encoding subunits of RNA polymerase I.

Supplementary Figure 9 Effect of NFAT5 ablation, of extended culture and transduction of KCs with control retroviruses (EGZ) or viruses expressing NFAT5-bio (N5) on the expression of “cornification proteins”. The effect of transduction of NFAT5 into tail-KCs (T) or KCs from newborn mice (NB) is shown.

Supplementary Figure 10 Effect of extended culture of KCs from tails of WT C57BL/6 mice, of NFAT5 ablation in 129/sv KCs and of transduction with a retrovirus expressing NFAT5 (N5) on keratin expression in tail KCs (T, left panels) or KCs from newborn mice (NB) (right panels). Data from at least two independent experiments are shown. Statistically different results are indicated by stars.

Supplementary Figure 11 Immunofluorescence stainings of sections through the skin of newborn mice (upper part) or of tails of adult mice (lower part). Sections were counterstained with DAPI and overlays are shown as indicated. The following Abs were used: NFAT5, ab110995; Mmp3, ab53015 (both Abcam). Length of the bars: 100 μm.

Supplementary Figure 12 Immunofluorescence co-stainings of sections through the skin of newborn (upper part) and adult mice (lower part). Sections were stained with Abs against NFAT5 (ab110995, Abcam) and Klk7 (Biotechn/R+D Systems, No.: AF2624) and counterstained with DAPI as indicated. The sections were counter- stained by DAPI. Length of the bars: 200 μm.

Supplementary Table 1 List of primers used in qRT-PCR assays.

Supplementary Table 2 List of TF genes whose expression was changed 2fold in KCs from tails of adult *Nfat5^-/-^* mice. Genes whose expression was enhanced 2fold and more: Gene whose expression was decreased 2fold and more.

## Abbreviations

Ab, antibody; KC, keratinocytes; Klk7, kallikrein 7; Mmp3, matrix metalloproteinase-3; NFAT, nuclear factor of activated T cells; NB mice, newborn mice; NGS, next generation sequencing; RPKM, reads per kilobase million; T, tail; TF, transcription factor; TPA, 12-O-tetradecanoylphorbol-13-acetate.
