# Supplementary figures and images for: NFAT5 Controls the Integrity of Epidermis
Source: Front Immunol. 2021 Dec 9;12:780727. doi: 10.3389/fimmu.2021.780727 (PMC8696207; doi:10.3389/fimmu.2021.780727)

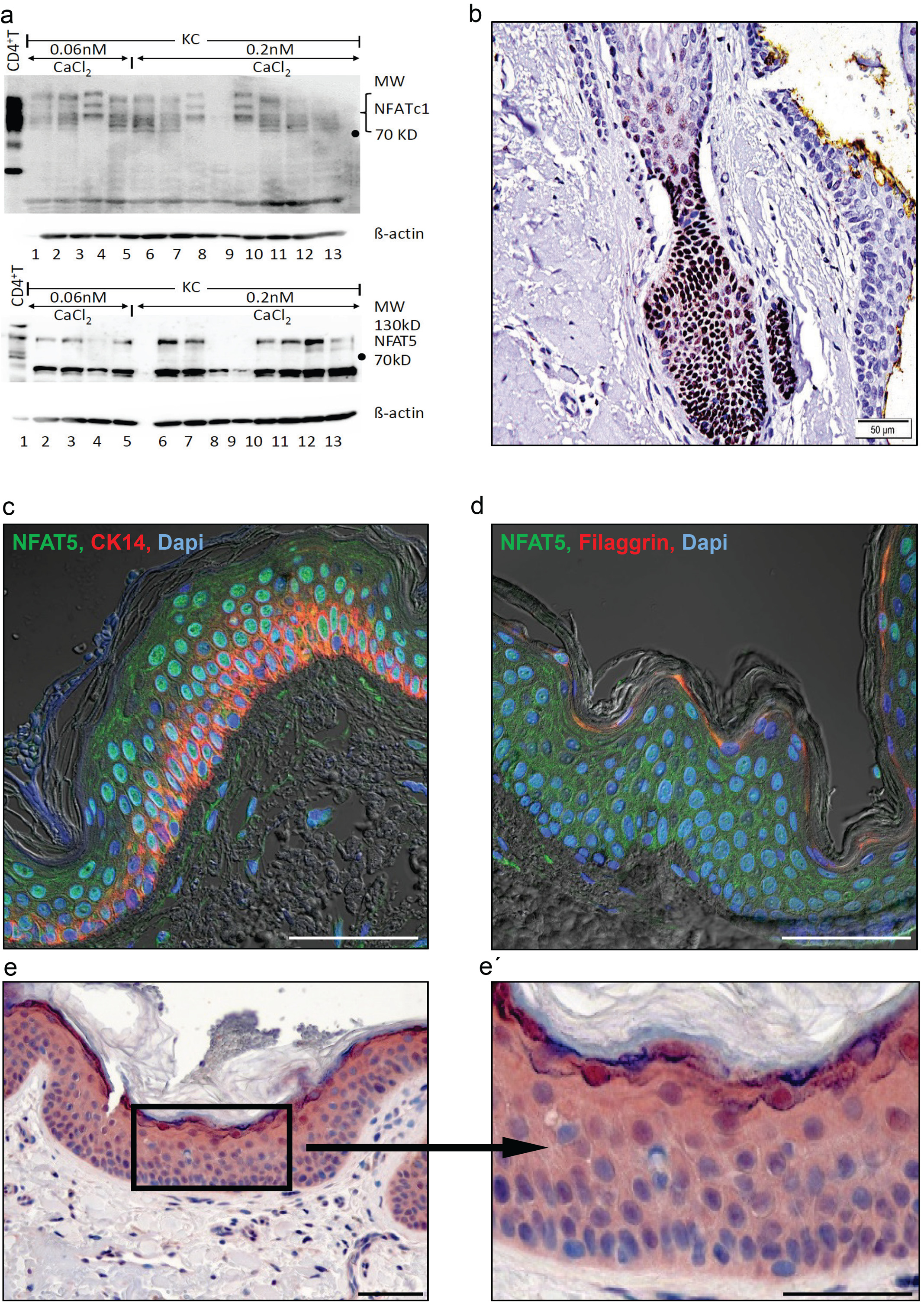

Supplement: Supplementary Figure 1 — NFAT5 and NFATc1 expression in epidermis of inter-follicular skin of mice and human. (a) Parallel Western blots with the same batches of whole cell protein extracts from primary murine KCs, compared to whole cell extracts from murine CD4+T cells. Upper blot, detection of NFATc1 expression by incubation with the NFATc1-specific mAb 7A6. Lane 1, murine CD4+ T cells activated by T+I for 24 h; lanes 2-5, KCs incubated for 3d in serum-free SFM KC medium (Gibco) containing 0.06 mM Ca++ without (lane 2) and with ionomycin (0.5 μM; lane 3), cyclosporine A (100 ng/ml; lane 4) or imiquimod (1 μg/ml; lane 5). In lanes 6-13, KCs were incubated in SFM medium containing 0.2 mM CaCl2 without (lane 6) and with ionomycin (lane 7), with cyclosporine A (lane 8), imiquimod (lane 9), ionomyin+cyclosporine A (lane 10) or ionomycin+imiquimod (lane 11). In lane 12 and 13, KCs were incubated in S-MEM medium containing 0.2 mM CaCl2 without (lane 12) or with ionomycin (lane 13). Lower blot, NFAT5 expression detected by pAb PAI-023 (Affinity Bio Reagents). For the lanes see (a). (b) Immuno-histochemical staining of a section through human skin stained with the NFATc1-specific mAb 7A6. (c) Co-staining of sections through human back skin with Abs specific for NFAT5 (ab3446) and cytokeratin 14 (sc-53253). (d) Co-staining of human back skin with Abs for NFAT5 (ab3446) and filaggrin (sc-66192). (e) Staining of human sun-exposed face skin with an NFAT5-specific Ab (sc-398171). An enlargement of framed part of (e) is shown in (é). Length of the bars: 50 μm. [file Image_1.jpeg]

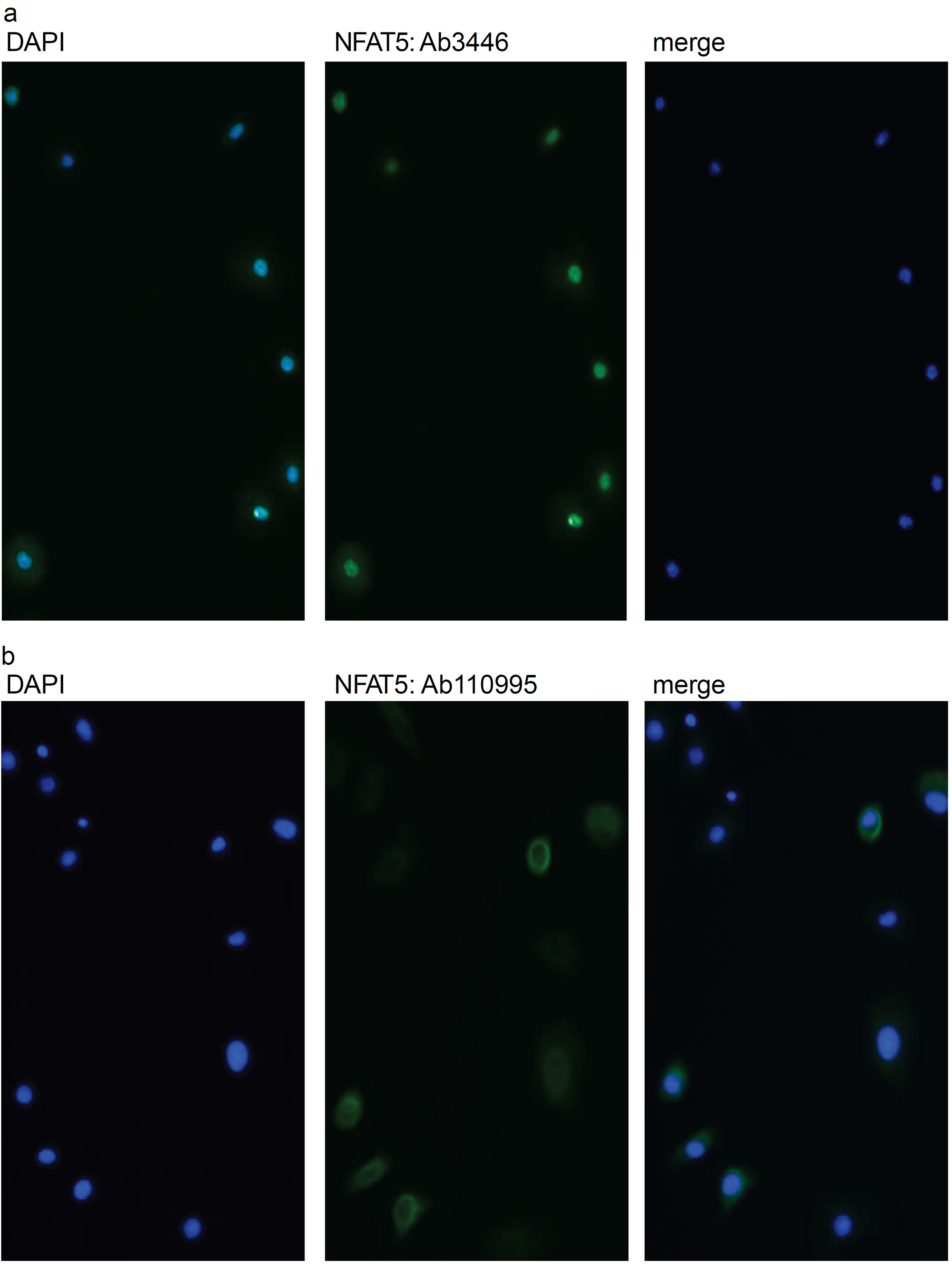

Supplement: Supplementary Figure 2 — Localization of NFAT5 in murine tail KCs upon culture for 1 week. Upon cytospin, the KCs were stained with the NFAT5-specific Abs ab3446 (a) or ab110995 (b). [file Image_2.jpeg]

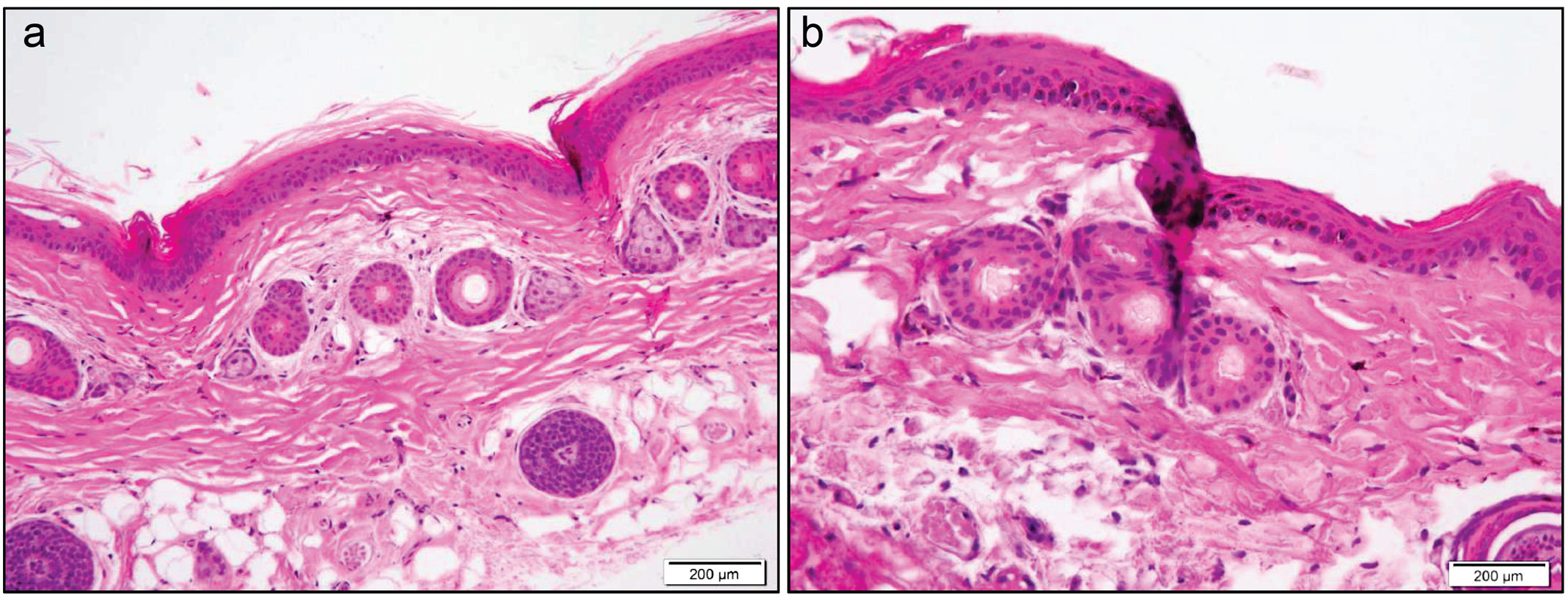

Supplement: Supplementary Figure 3 — Representative H&E stains of sections through the skin from tails of WT129/sv (a) and Nfat5-/- 129/sv mice (b). Note the missing corneocyte layer in skin from Nfat5-/- mice. [file Image_3.jpeg]

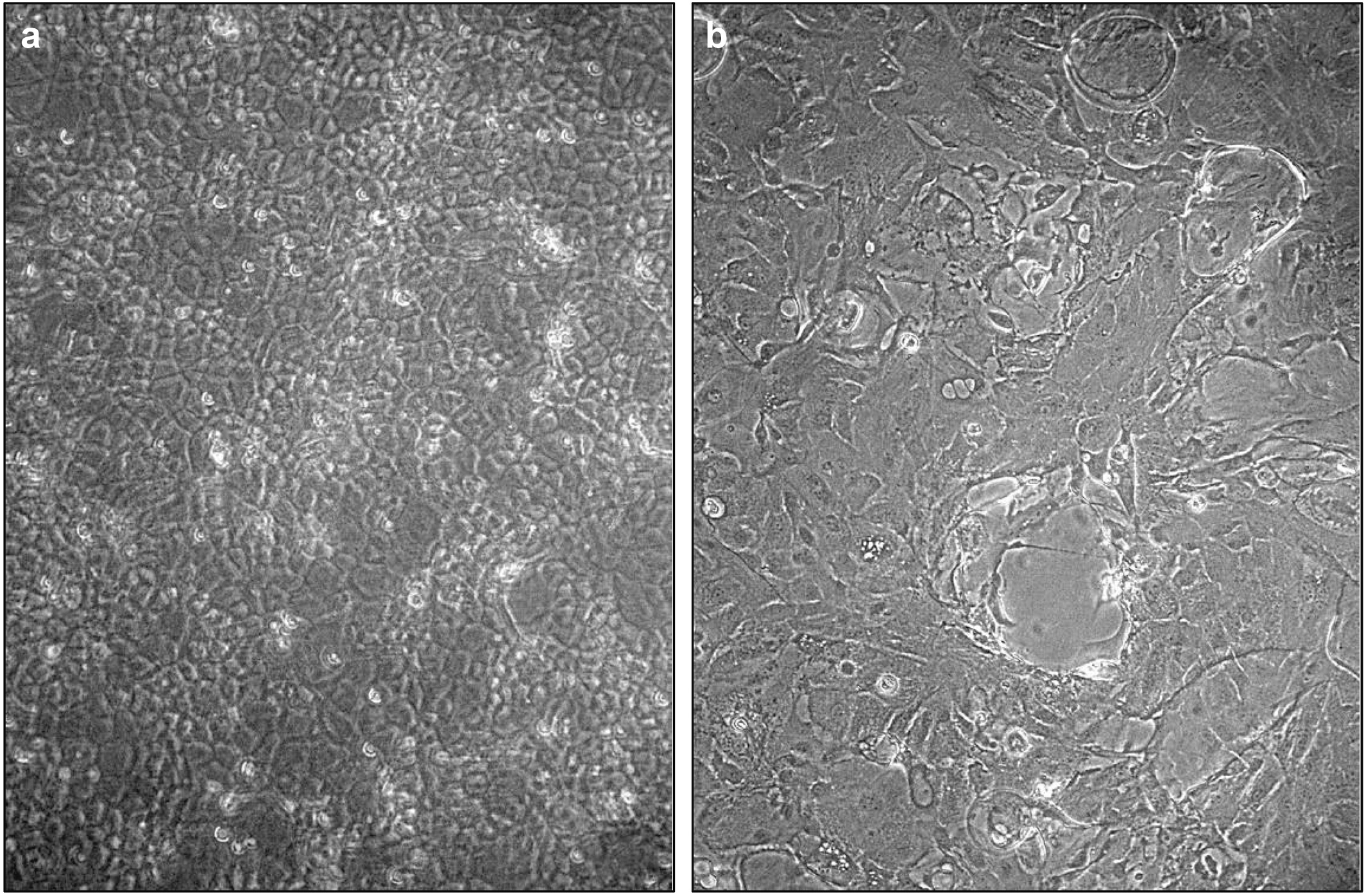

Supplement: Supplementary Figure 4 — Primary KCs from tails of WT C57/Bl6 mice cultured for one week (a) or three weeks (b) in vitro. [file Image_4.jpg]

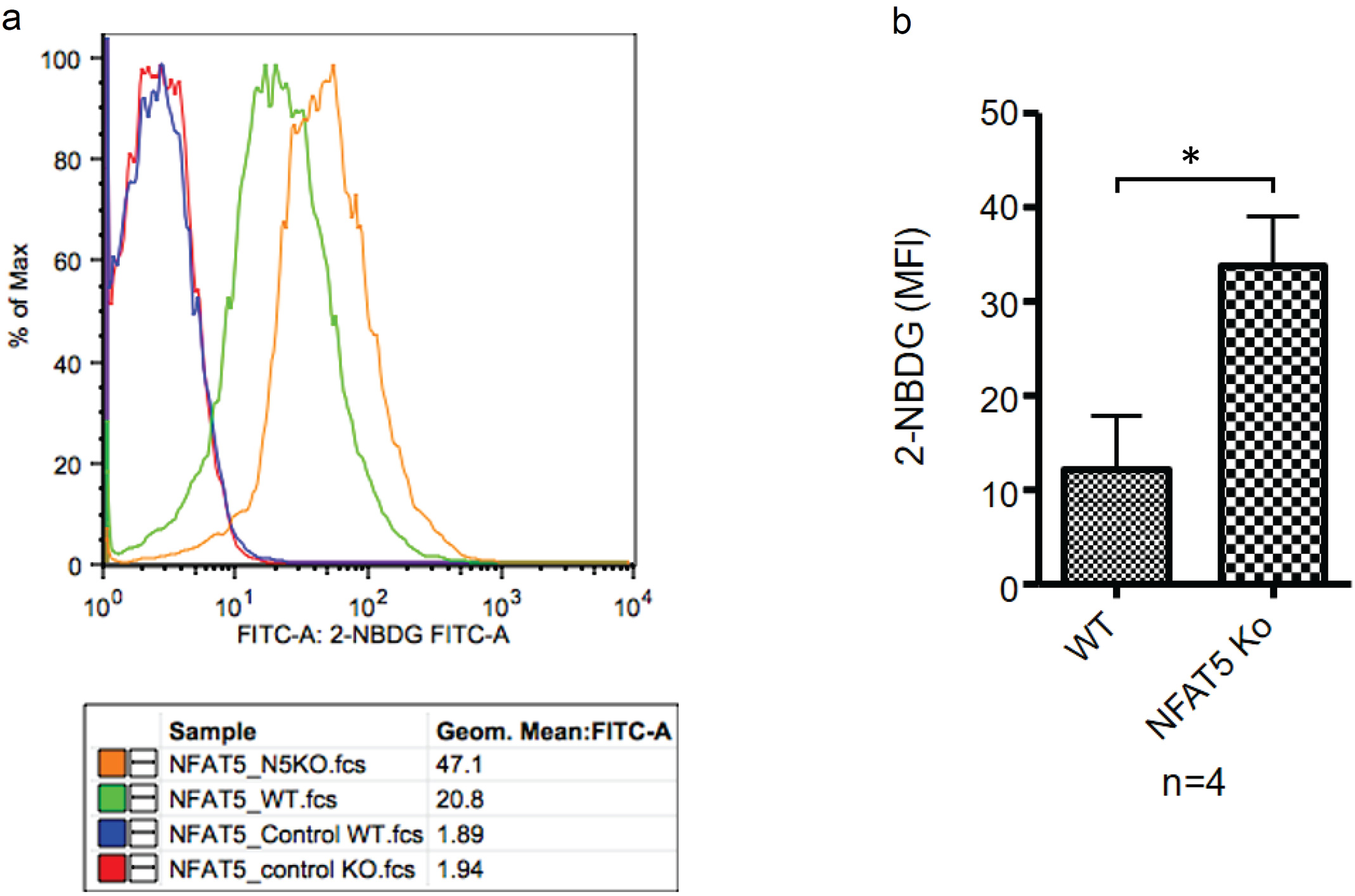

Supplement: Supplementary Figure 5 — Increased uptake of the fluorescent glucose analog 2-NBDG (2-[N-(7-nitrobenz-2-oxa-1,3-diazol-4-yl) amino]-2deoxy-D-glucose) into NFAT5-deficient 129/sv KCs, as compared to WT 129/sv KCs. (a) Flow cytometry of FITC-labeled 2-NBDG, and (b) quantification of 2-NBDG uptake. [file Image_5.jpeg]

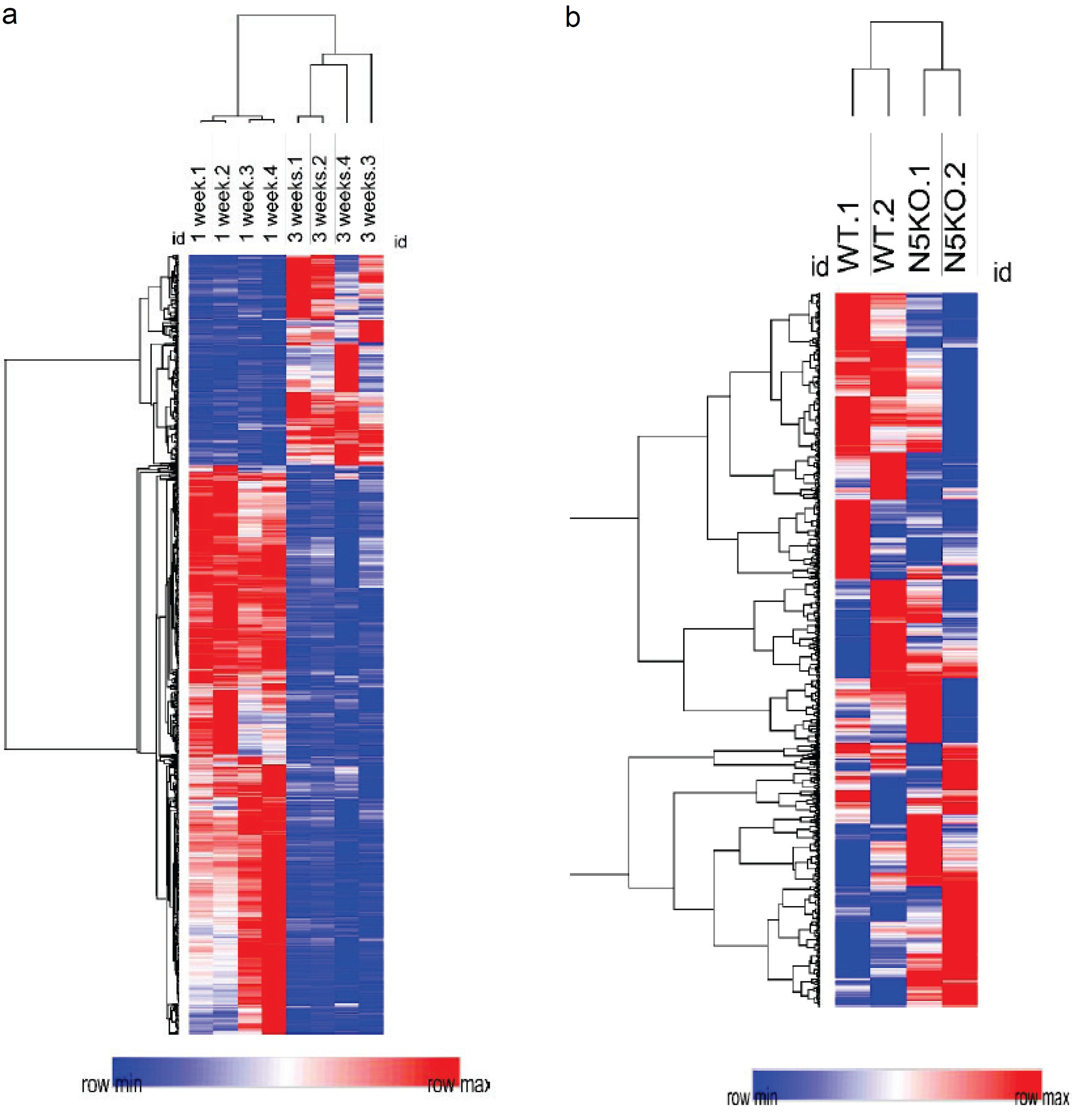

Supplement: Supplementary Figure 6 — Heat maps of more than 8400 genes that were expressed in 3 and more RPKMs in basal murine KCs. (a) Transcriptomes of KCs from WT C57BL/6 mice cultured for one or three weeks in vitro. Data from four independent experiments were compiled with cells being cultured for either 1 or 3 weeks. (b) Heat maps of transcriptomes from KCs of 129/sv mice. The two WT and Nfat5-/- KC cultures were maintained in vitro for 1 week. [file Image_6.jpeg]

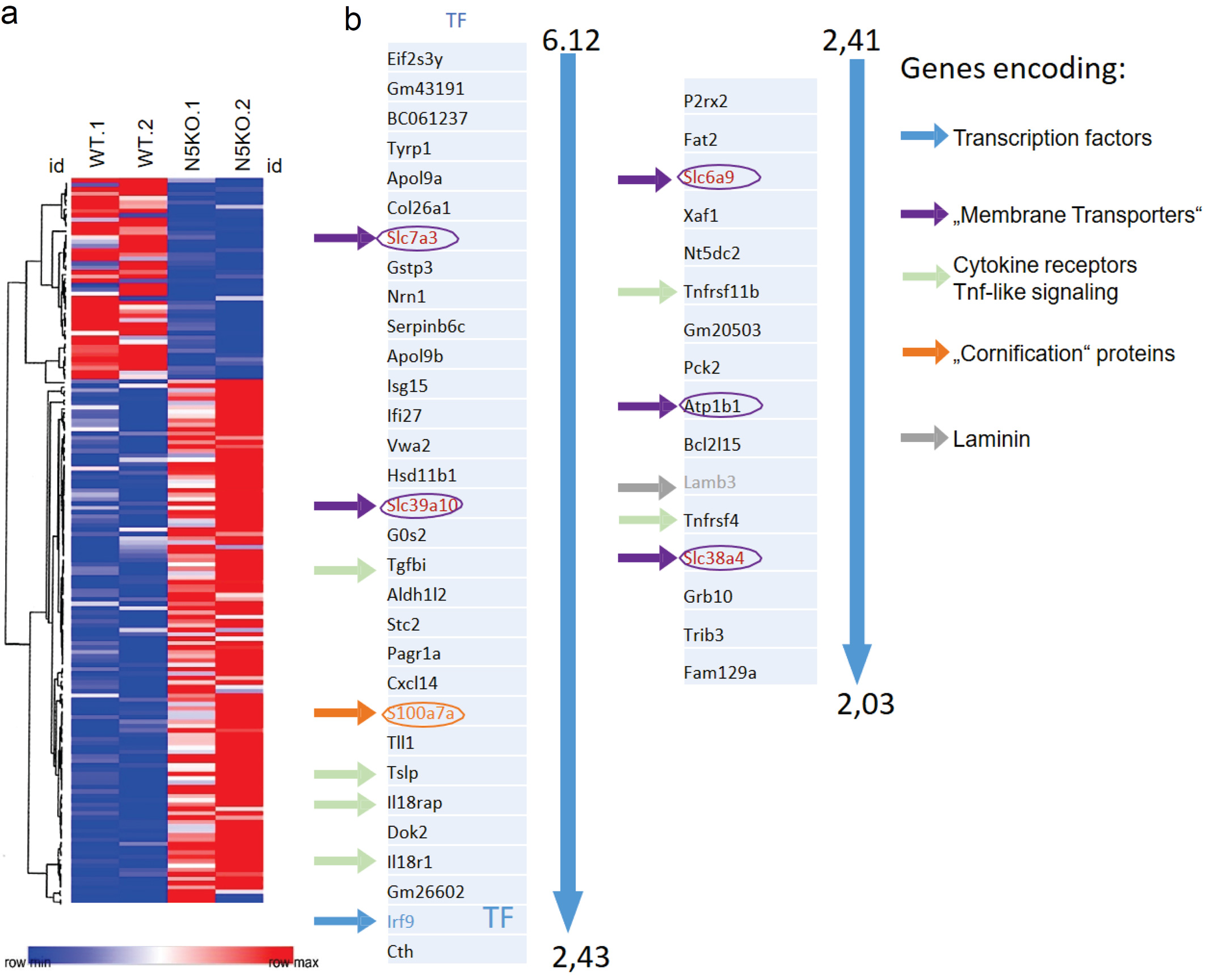

Supplement: Supplementary Figure 7 — Compilation of genes that were 2fold weaker expressed in Nfat5-/- than WT 129/sv KCs. (a) Heat map of 168 genes that were changed at least 2fold in expression between WT and Nfat5-/- KCs. (b) Compilation of 47 genes that were expressed in 2fold less copies in Nfat5-/- KCs. The gene encoding the TF Irf9 is marked by a blue arrow. Five genes encoding membrane transporters are highlighted by violet arrows. Genes coding for cytokine receptors and Tnf-signaling molecules are indicated by green arrows, and the laminin gene Lamb3 and the gene encoding S1007a, a “cornification protein”, are indicated by a grey or red arrows, respectively. [file Image_7.jpeg]

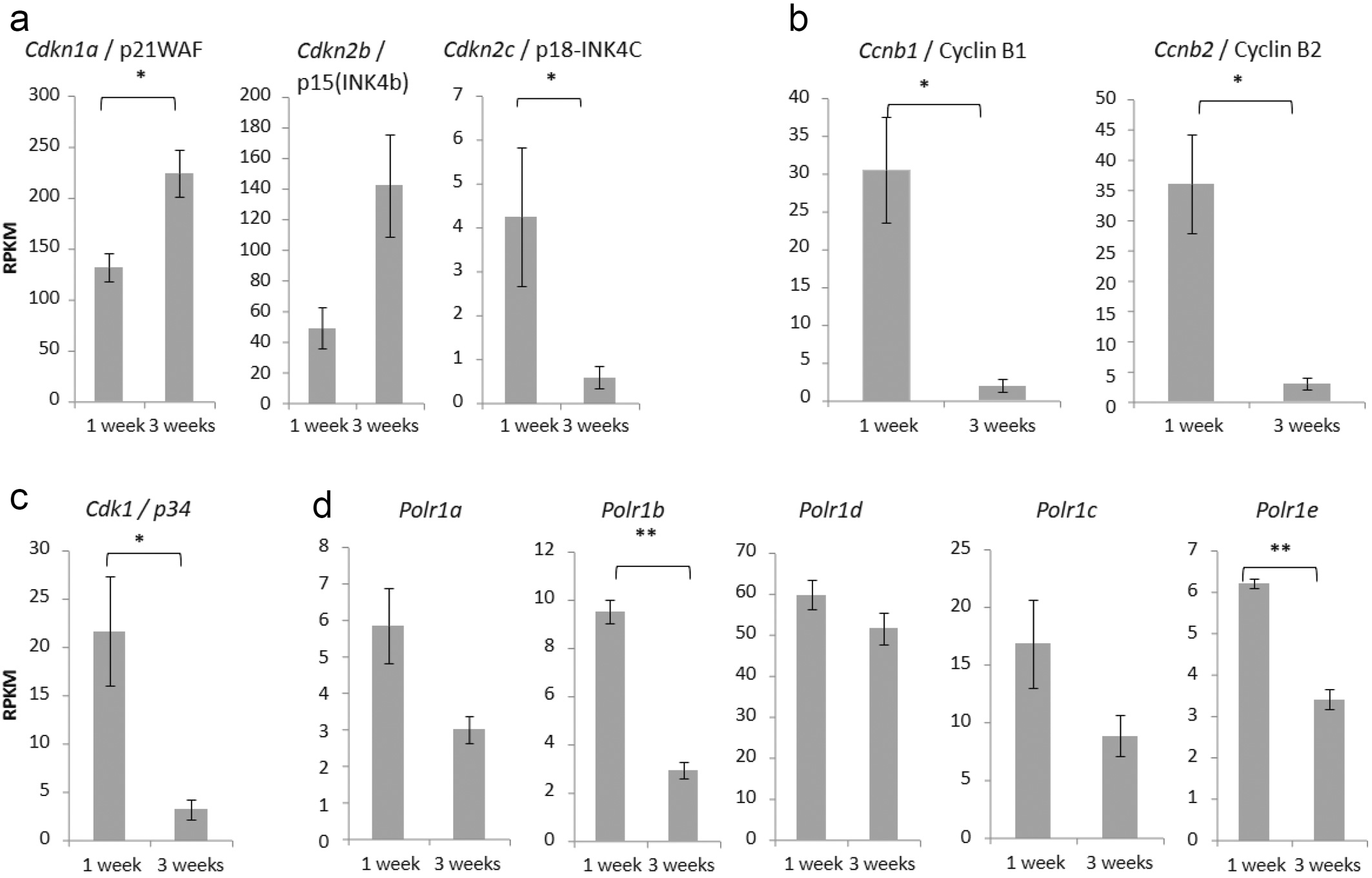

Supplement: Supplementary Figure 8 — Extended culture of WT C57BL/6 KCs affects the expression of genes that control cell cycle. (a) Genes encoding cell cycle inhibitors. (b) Cyclin genes. (c) Cyclin-dependent kinase Cdk1 gene. (d) Genes encoding subunits of RNA polymerase I. [file Image_8.jpeg]

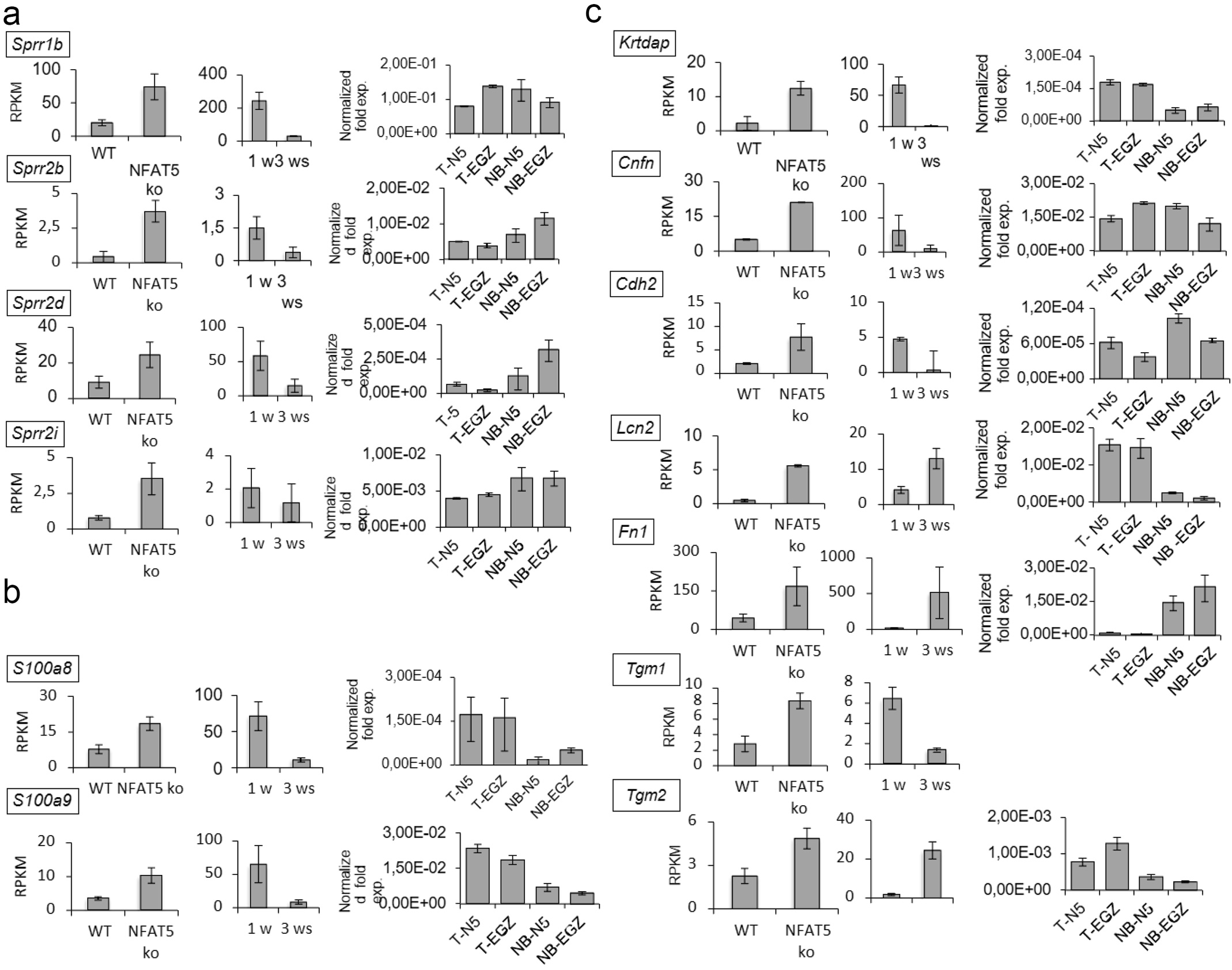

Supplement: Supplementary Figure 9 — Effect of NFAT5 ablation, of extended culture and transduction of KCs with control retroviruses (EGZ) or viruses expressing NFAT5-bio (N5) on the expression of “cornification proteins”. The effect of transduction of NFAT5 into tail-KCs (T) or KCs from newborn mice (NB) is shown. [file Image_9.jpeg]

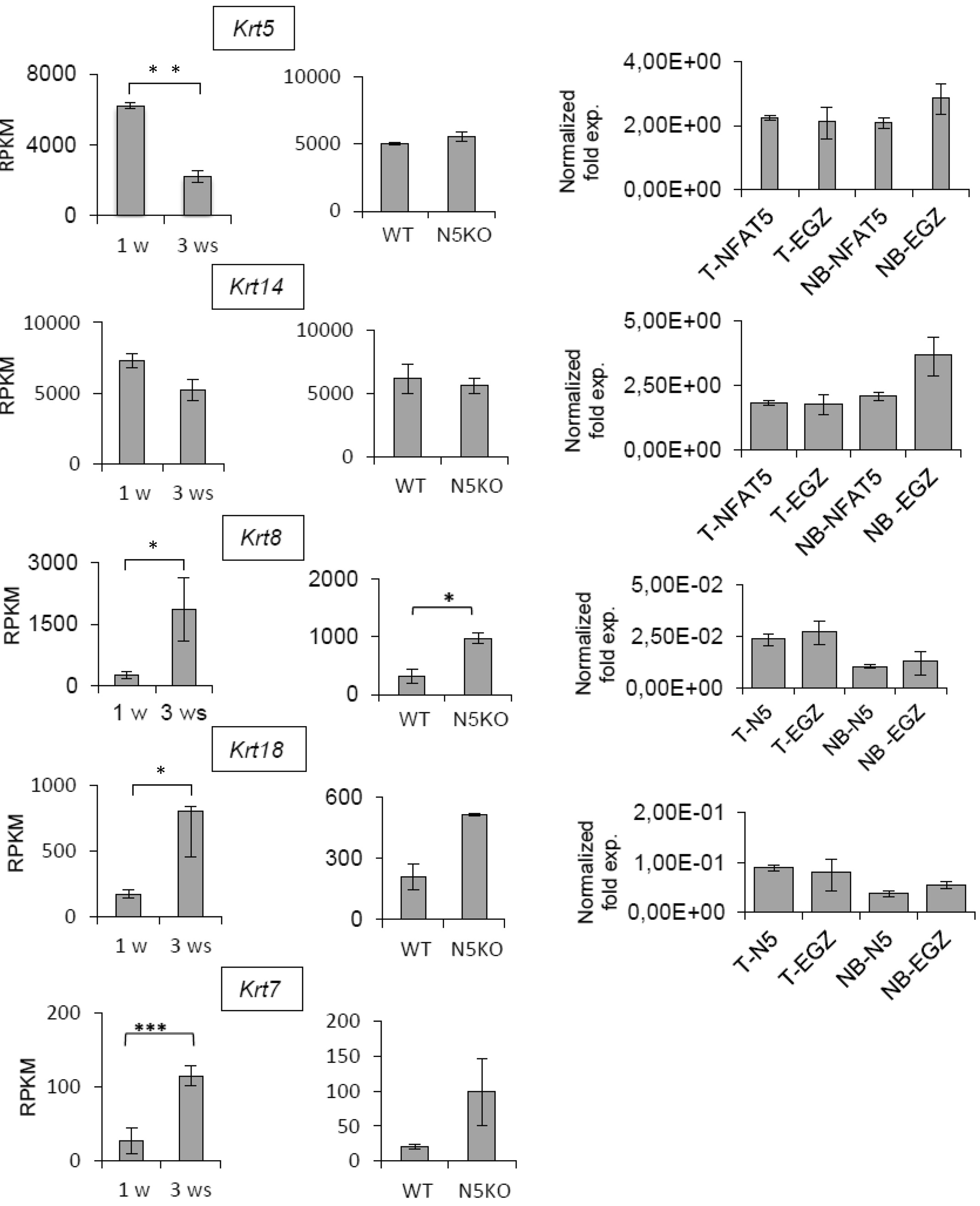

Supplement: Supplementary Figure 10 — Effect of extended culture of KCs from tails of WT C57BL/6 mice, of NFAT5 ablation in 129/sv KCs and of transduction with a retrovirus expressing NFAT5 (N5) on keratin expression in tail KCs (T, left panels) or KCs from newborn mice (NB) (right panels). Data from at least two independent experiments are shown. Statistically different results are indicated by stars. [file Image_10.jpeg]

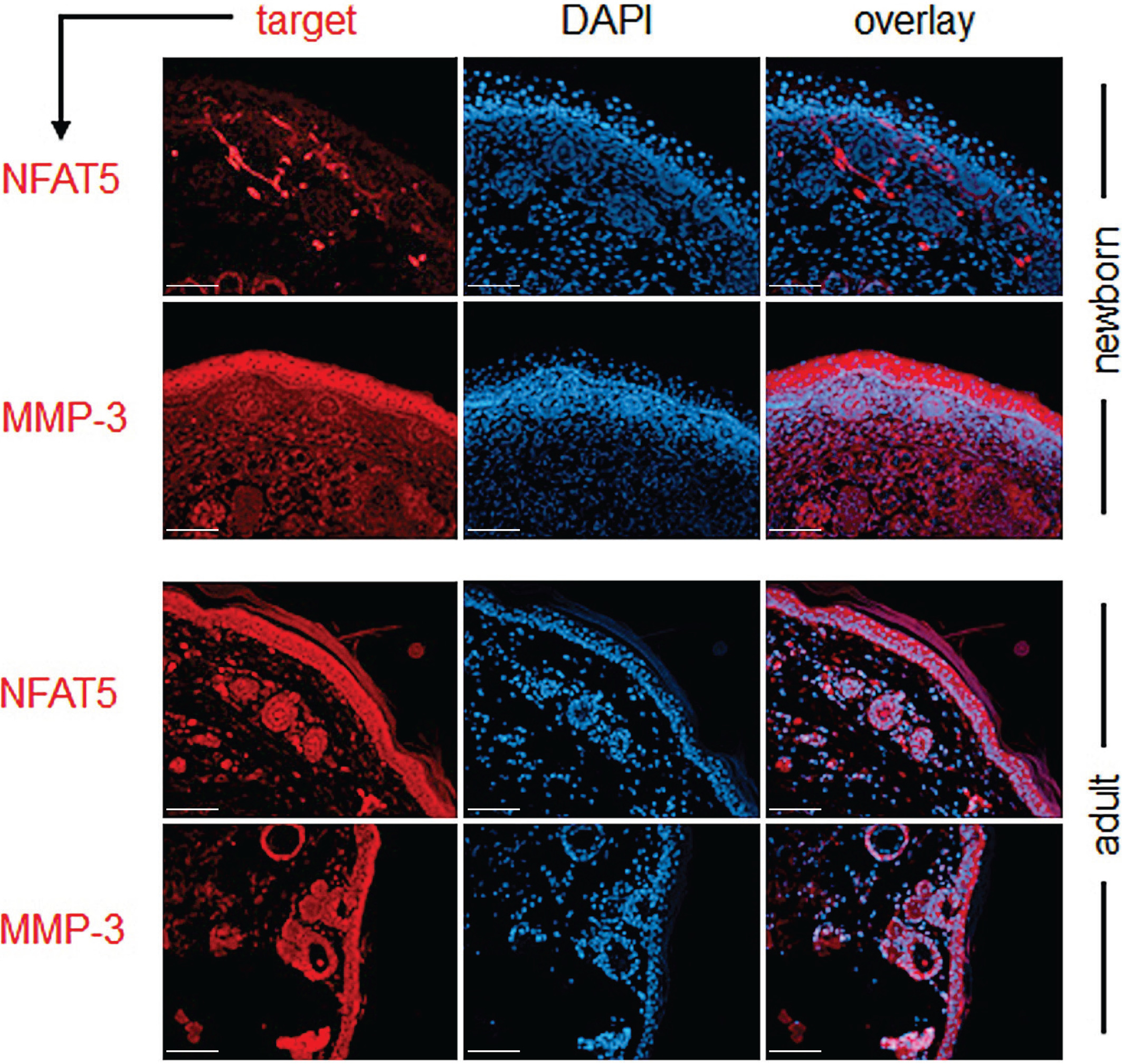

Supplement: Supplementary Figure 11 — Immunofluorescence stainings of sections through the skin of newborn mice (upper part) or of tails of adult mice (lower part). Sections were counterstained with DAPI and overlays are shown as indicated. The following Abs were used: NFAT5, ab110995; Mmp3, ab53015 (both Abcam). Length of the bars: 100 μm. [file Image_11.jpeg]

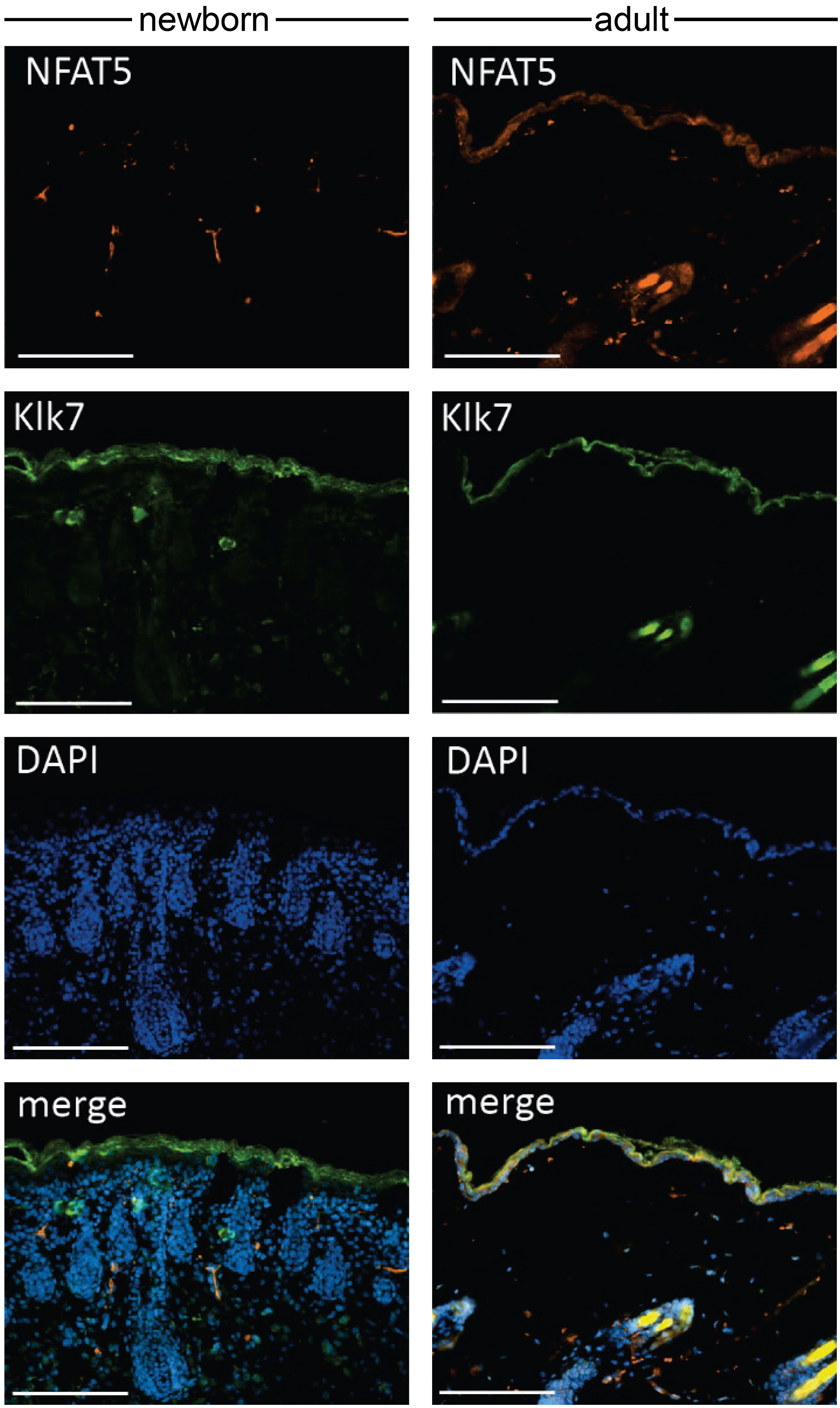

Supplement: Supplementary Figure 12 — Immunofluorescence co-stainings of sections through the skin of newborn (upper part) and adult mice (lower part). Sections were stained with Abs against NFAT5 (ab110995, Abcam) and Klk7 (Biotechn/R+D Systems, No.: AF2624) and counterstained with DAPI as indicated. The sections were counter- stained by DAPI. Length of the bars: 200 μm. [file Image_12.jpeg]
