# Supplementary material for: NFAT5 Controls the Integrity of Epidermis
Source: Front Immunol. 2021 Dec 9;12:780727. doi: 10.3389/fimmu.2021.780727 (PMC8696207; doi:10.3389/fimmu.2021.780727)
Supplement: Supplementary Table 1 — List of primers used in qRT-PCR assays. [file Table_1.pdf]

Supplementary Materials

Supplementary Table 1. List of primers used in qRT-PCR assays

**Genes**	Forward	Reverse
ActB	CTCTGGCTCCTAGCACCATGAAGA	GTAAAACGCAGCTCAGTAACAGTCCG
B2m	CTGCTACGTAACACAGTTCCACCC	CATGATGCTTGATCACATGTCTCG
Asprv1	ATGCCCTGGATGTCTACAAT	CATGCTGTTGGCAAACAAAA
Cnfn	TGAGAGTTCGTCTCCCCCAG	ACTGGATATGACATGGCTTTGC
Cdh2	CACGCACACGCGTGCACACA	CGGACTCGCACCAGGAGTAA
Cdkn2a	TGGTCACTGTGAGGATTCAGC	TTGCCCATCATCATCACCTGG
Mmp3	GTCCCTCTATGGAACTCCCAC	AGTCCTGAGAGATTTGCGCC
Fn1	CGAAGAGCCCTTACAGTTCCA	ATCTGTAGGCTGGTTCAGGC
Sprr1b	TGTCCTCCATATACCAGGCTCA	GAGACACATGGCTCAGGAGG
Sprr2d	CCGAGACTACTTTGGAGAACCC	GGCTCTGGGCATTTTGGA
S100a9	TTTAGCCTTGAGCAAGAAGATGG	AGGGTGTCAGGGTGTCCTTC
S100a8	TGACTTCAAGAAAATGGTCA	CCACTTTTATCACCATCGCA
Sprr2b	TGGCAGCCAAAATGCCCTCC	TGTTGCTATGGAGTTGGTGA
Krtdap	AACAGAGGGCCTTAACAATG	TAGGGAAGAAGTCCCAGTTA
Sprr2d	TGTCCTGAGCCATGTCCCCC	ATTCTCTTCTTTTCCCTTTG
Sprr2i	TACCAGCAGAAATGCCCTCC	TTGTTGCTATGGAGGCATGT
Tgm1	CGGGGTGGCCGAAGACCCGA	GTGTGGTGCTCTCGGCGGTT
Tgm2	CGGCCCAGATCCCAGTGAAG	CATTGGCTGGGGTGCAGAGC
Krt5	TCTGCCATCACCCCATCTGT	CCTCCGCAGAACTGTAGGA
Krt14	AGCGGCAAGAGTGAGATTTCT	CCTCCAGGTTATTCTCCAGGG
Lcn2	ATGTCACCTCCATCCTGGTCAG	GCCACTTGCACATTGTAGCTCTG
Klk7	TGCAAAATGCGTCAGTACCA	GACCTGGGTCTTTGTGGAGT
Krt8	TGGAAGGACTGACCGACGAGAT	GGCACGAACTTCAGCGATGATG
Krt18	AATCAGGGACGCTGAGACCACA	GCTCCATCTGTGCCTTGTATCG
