# Supplementary material for: NFAT5 Controls the Integrity of Epidermis
Source: Front Immunol. 2021 Dec 9;12:780727. doi: 10.3389/fimmu.2021.780727 (PMC8696207; doi:10.3389/fimmu.2021.780727)
Supplement: Supplementary Table 2 — List of TF genes whose expression was changed 2fold in KCs from tails of adult Nfat5-/- mice. Genes whose expression was enhanced 2fold and more: Gene whose expression was decreased 2fold and more. [file Table_2.pdf]

Supplementary Table 2. List of TF genes whose expression was changed 2fold in KCs from tails of adult *Nfat5*$^{-/-}$ mice.

Genes whose expression was enhanced 2fold and more:

Gene	Protein	Function in Epidermis/Skin	Refs.
Barx2	BarH-like homeobox 2 Factor	Transcriptional repressor that regulates hair follicle remodelling.	(37)
Foxn1	Forkhead box N1<br>Whn	Nude mice: skin disorder congenital alopecia, and nail dystrophy.<br>Foxn1 drives skin healing via engagement in re-epithelization and the epithelial-mesenchymal transition (EMT) process (Mmp9 as target gene).	(39)<br>(50)
Mxd1	MAX dimerization protein 1<br>MAD1	Antagonizes Myc activity. Highly expressed in epidermal KCs.<br>Mad:Max complexes are detected during differentiation and appear to replace the Myc:Max complexes present in proliferating keratinocytes and other cells.	(33)
Sox11	SRY-box TF11	Dictates embryonic epidermal state, reactivation of embryonic genes during wound repair.<br>Peripheral neuron regeneration.	(40)
Hopx	HOP homeobox	Transcriptional co-factor, does not bind to DNA.<br>Positive regulator of late KC differentiation.<br>Marker for a subset of multipotent hair follicle stem cells.	(34)
Grhl3	Grainyhead like transcription factor 3	Mouse skin lacking Grhl3 showed increased infiltration of mast cells and pro-inflammatory T cells, increased expression of the pro-proliferative/pro-inflammatory markers CD3 and pSTAT3, and significantly elevated basal KC proliferation.<br>GRHL3 regulates barrier formation during epidermal development.	(35)
Klf10	Krüppel-like factor 10	Suppressor of KC proliferation and apoptosis.<br>p21^{WAF} is a downstream target. Tumor-Suppressor.	(36)
Egr1	Early growth response 1	Upregulation in psoriatic skin lesions by IL-17A, binding to the psoriasin (S100A7) promoter.<br>Early induced gene in wounded KCs.	(42)
Zfp750	Zinc finger protein 750	Role inf seborrhea-like dermatitis with psoriasiform elements. Selective expression in KCs.	(43)

Gene whose expression was decreased 2fold and more:

Irf9	Interferon regulatory factor 9	Highly expressed in psoriatic lesions of skin	(44)
